# Degenerated Hole Doping and Ultra‐Low Lattice Thermal Conductivity in Polycrystalline SnSe by Nonequilibrium Isovalent Te Substitution

**DOI:** 10.1002/advs.202105958

**Published:** 2022-03-08

**Authors:** Xinyi He, Haoyun Zhang, Takumi Nose, Takayoshi Katase, Terumasa Tadano, Keisuke Ide, Shigenori Ueda, Hidenori Hiramatsu, Hideo Hosono, Toshio Kamiya

**Affiliations:** ^1^ Laboratory for Materials and Structures, Institute of Innovative Research Tokyo Institute of Technology 4259 Nagatsuta, Midori Yokohama 226‐8503 Japan; ^2^ Research Center for Magnetic and Spintronic Materials National Institute for Materials Science 1‐2‐1 Sengen Tsukuba Ibaraki 305‐0047 Japan; ^3^ Research Center for Functional Materials National Institute for Materials Science Namiki Tsukuba 305‐0044 Japan; ^4^ Research Center for Advanced Measurement and Characterization National Institute for Materials Science Tsukuba 305‐0047 Japan; ^5^ Synchrotron X‐ray Station at SPring‐8 National Institute for Materials Science 1‐1‐1 Sayo Hyogo 679‐5148 Japan; ^6^ Materials Research Center for Element Strategy Tokyo Institute of Technology 4259 Nagatsuta, Midori Yokohama 226‐8503 Japan

**Keywords:** defect, phonon transport, solid solution, thermoelectric materials, tin mono‐selenide

## Abstract

Tin mono‐selenide (SnSe) exhibits the world record of thermoelectric conversion efficiency *ZT* in the single crystal form, but the performance of polycrystalline SnSe is restricted by low electronic conductivity (*σ*) and high thermal conductivity (*κ*), compared to those of the single crystal. Here an effective strategy to achieve high *σ* and low *κ* simultaneously is reported on p‐type polycrystalline SnSe with isovalent Te ion substitution. The nonequilibrium Sn(Se_1−_ *_x_* Te*_x_*) solid solution bulks with *x* up to 0.4 are synthesized by the two‐step process composed of high‐temperature solid‐state reaction and rapid thermal quenching. The Te ion substitution in SnSe realizes high *σ* due to the 10^3^‐times increase in hole carrier concentration and effectively reduced lattice *κ* less than one‐third at room temperature. The large‐size Te ion in Sn(Se_1−_ *_x_* Te*_x_*) forms weak Sn—Te bonds, leading to the high‐density formation of hole‐donating Sn vacancies and the reduced phonon frequency and enhanced phonon scattering. This result—doping of large‐size ions beyond the equilibrium limit—proposes a new idea for carrier doping and controlling thermal properties to enhance the *ZT* of polycrystalline SnSe.

## Graphical abstract

Synergistic heavy hole doping and thermal conductivity reduction are demonstrated in polycrystalline SnSe by nonequilibrium isovalent Te ion substitution. The weak Sn—Te bonds formed in layered Sn(Se_1−_ *_x_* Te*_x_*) doped with large‐size Te ions not only increases the hole‐donating Sn vacancies but also enhances the phonon scattering, resulting in the degenerate hole conduction and ultra‐low lattice thermal conductivity of SnSe.

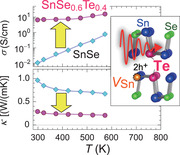

## 1 Introduction

Due to the recently growing energy issues, the importance of thermoelectric energy conversion, which directly converts waste heat into electricity, has been increasing as a potential energy harvesting technology. The conversion efficiency is generally qualified in terms of a dimensionless figure of merit, *ZT*, where *Z* = *S* ^2^ × *σ* × *κ* ^−1^ is the figure of merit, *T* is the absolute temperature, *S* is the Seebeck coefficient, *σ* is the electronic conductivity, and *κ* is the sum of the electronic (*κ*_ele_) and lattice thermal conductivities (*κ*_lat_) of a thermoelectric material.^[^ ^1^, ^2^ ^]^

Tin mono‐selenide (SnSe) with a layered crystal structure has extensively been studied as a potential thermoelectric material since the discovery of the world‐record *ZT* up to 2.6 in the single crystal at high *T* of 926 K.^[^ ^3^ ^]^ Pure SnSe is an indirect‐gap semiconductor with a band gap of ≈1.0 eV^[^ ^4^, ^5^, ^6^, ^7^, ^8^ ^]^ and shows weak p‐type conduction by the hole‐donating Sn vacancies (*V*_Sn_).^[^ ^9^ ^]^ The high *ZT* is attributed to ultra‐low *κ*_lat_ due to giant phonon anharmonicity in the layered structure.^[^ ^10^, ^11^, ^12^, ^13^ ^]^ The SnSe single crystals exhibit highly anisotropic thermoelectric properties and the remarkable *ZT* is realized along the in‐layer direction. On the contrary to high *ZT* in the single crystal, the thermoelectric performance of polycrystalline SnSe was significantly decreased to *ZT* ≈ 0.5 even at high *T* = 823 K^[^ ^14^ ^]^ because of its lower *σ* and higher *κ*_lat_ than those of the single crystal. Performance enhancement of polycrystalline SnSe has been a key challenge for their practical applications.

To date, two major strategies for achieving high *ZT* in SnSe polycrystals are maximizing the power factor (PF = *S* ^2^ *σ*) by the aliovalent ion substitution and reducing *κ*_lat_ by nano‐structuring. *σ* and *S* are functions of carrier concentration (*n*), where the *σ* increases with growing *n* whereas *S* decreases. Thus, PF can be maximized only if *n* is fully optimized. The pure SnSe has low hole *n* ≈ 10^15^–10^17^ cm^−3^,^[^ ^14^, ^15^, ^16^, ^17^ ^]^ while monovalent alkali ions and Ag^+^ ion substitution at the Sn^2+^ site works as an effective acceptor, which realized high hole *n* up to 10^19^ cm^−3^ at room temperature (RT) to achieve a high PF, and resulted in an enhanced *ZT* over a wide *T* range.^[^ ^18^, ^19^, ^20^, ^21^ ^]^ The maximum *ZT* was increased up to ≈0.8 at high *T* = 800 K for Na 1% doped SnSe.^[^ ^21^ ^]^ Even for that, the *ZT* has been limited to < ≈0.3 at low *T* ≤ 573 K. Therefore, the additional nano‐structuring process, that is, the addition of precipitates such as Ag_8_SnSe_6_, PbSe, and carbon fiber,^[^ ^22^, ^23^, ^24^ ^]^ has been adopted to introduce phonon scattering centers to reduce *κ*_lat_, which realized the higher *ZT* ≈ 1.3 at high *T* = 773–823 K and ≈0.6 at low *T* = 573 K in polycrystalline SnSe. Thus, the well improved thermoelectric SnSe usually needs complicated processes not only for a fine optimization of *n* and PF, but also a selective decrease of *κ*_lat_ in order to maximize the *ZT*.

**Figure 1:**
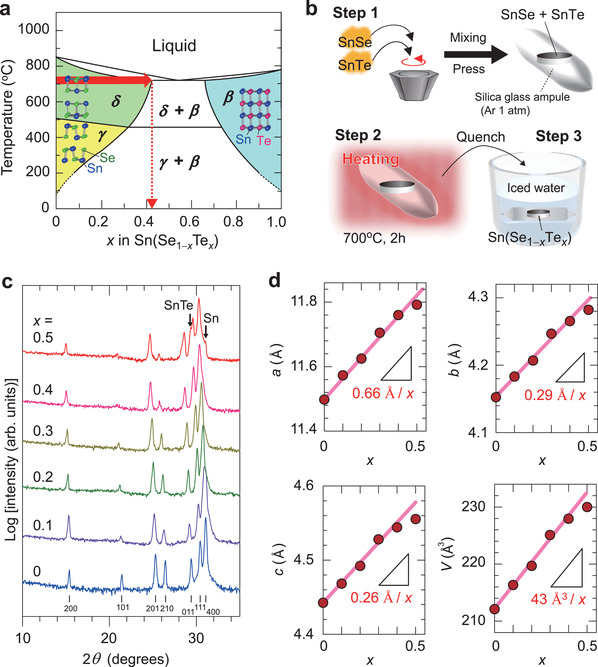
Synthesis of non‐equilibrium Sn(Se_1−_ *_x_* Te*_x_*) solid solution bulks. a) Phase diagram of SnSe‐SnTe system modified from ref. [30]. SnSe has an orthorhombic GeS‐type layered crystal structure (*γ* phase, space group: *Pnma*) as thermodynamically stable phase at *T* < 500 °C. SnSe transforms to orthorhombic *Cmcm* phase (*δ* phase) at higher *T* > 500 °C. The layered structure is composed of the alternately stacked single‐molecular SnSe layers with threefold coordinated Sn‐Se_3_. The counterpart SnTe has the rock‐salt type cubic structure (*β* phase, space group: *Fm* $\bar{3}$ *m*) with sixfold coordinated Sn‐Te_6_. b) Schematic procedure of the two‐step non‐equilibrium processes combining high‐*T* solid‐state reaction and rapid quenching, which is effective to stabilize the high‐temperature layered Sn(Se_1−_ *_x_* Te*_x_*) solid solution phase down to RT, as indicated by the dotted red arrow in (a). c) XRD patterns of Sn(Se_1−_ *_x_* Te*_x_*) bulk polycrystals with *x* = 0−0.5 at RT. The vertical bars at bottom denote the diffraction angles of the layered SnSe phase (*γ* phase, *Pnma*). The arrows for *x* = 0.5 indicate the diffraction peaks from SnTe and Sn impurities. d) *a*‐, *b*‐, and *c*‐axes lattice parameters (*a*, *b*, and *c*) and unit cell volume (*V*) as a function of *x*.

In this work, we report that isovalent Te ion substitution is an effective approach for simultaneous increase of PF and decrease of *κ*_lat_ in polycrystalline SnSe without nano‐structuring. There has been a contradiction in the previously reported properties of Sn(Se_1−_ *_x_* Te*_x_*) bulks, for example, lightly Te‐doped SnSe_0.94_Te_0.06_ exhibits n‐type conductivity,^[^ ^25^ ^]^ heavily Te‐doped SnSe_0.8_Te_0.2_ exhibits *σ* smaller than that of the pristine p‐type SnSe,^[^ ^26^ ^]^ while another paper reports Te doping up to *x* = 0.2 increases p‐type conductivity.^[^ ^27^ ^]^ The difficulty comes from the low solubility limit of *x* in Sn(Se_1−_ *_x_* Te*_x_*);^[^ ^28^, ^29^ ^]^ as found in the SnSe−SnTe phase diagram (**Figure** **1**a),^[^ ^30^ ^]^ the isovalent Te^2−^ ions can substitute a part of the Se^2−^ sites in the layered SnSe (*δ* and *γ* phases), but the solubility limit of Te ion is extremely low under thermal equilibrium presumably because of the large ion‐size mismatch between Se^2−^ (ionic radius of 1.98 Å) and Te^2−^ (2.21 Å), as well as their different coordination structures of the layered SnSe with threefold coordination and the counterpart cubic SnTe (*β* phase) with sixfold coordination. Actually, as seen in the phase diagram in Figure 1a, the phase separation to the layered and the cubic phases (‘*δ* + *β*’ and ‘*γ* + *β*’) occurs by the simple solid‐state reaction at thermal equilibrium.^[^ ^31^ ^]^

We herein overcome the solubility limit problem in Sn(Se_1−_ *_x_* Te*_x_*) by the two‐step nonequilibrium growth process composed of high‐*T* solid‐state reaction and rapid thermal quenching (Figure 1b), in order to stabilize the layered Sn(Se_1−_ *_x_* Te*_x_*) solid solution phase at high *T* of 700 °C to RT, as indicated by the red arrows in Figure 1a. We succeeded to expand the Te concentration *x* in the layered Sn(Se_1−_ *_x_* Te*_x_*) bulk polycrystals up to *x* = 0.4. Usually isovalent ion doping does not work for free carrier doping to ionic semiconductors. We, however, found that isovalent Te^2−^ ion substitution at the Se^2−^ site in SnSe unusually increases *n* by three orders of magnitude and also largely reduces *κ* from 1.0 W m^−1^K^−1^ of pure SnSe down to 0.3 W m^−1^K^−1^ at RT. This work clarifies the impact of the isovalent Te ion substitution both in the electrical transport and thermal transport properties of SnSe with an assistance of the first‐principles calculations.

## 2 Results and Discussion

### 2.1 Synthesis of Non‐Equilibrium Sn(Se_1−_ *_x_* Te*_x_*) Bulk

Figure 1c shows the logarithmic‐scaled X‐ray diffraction (XRD) patterns of polycrystalline Sn(Se_1−_ *_x_* Te*_x_*) bulks with *x* = 0–0.5. The chemical compositions (i.e., the atomic ratio of Sn, Se, and Te) of the bulk samples were measured with X‐ray fluorescence spectroscopy (Figure S1, Supporting Information). The main crystalline phase of all the samples is assigned to the orthorhombic layered SnSe (*γ* phase, space group: *Pnma*), where impurities of cubic SnTe (*Fm* $\bar{3}$ *m*) and tetragonal Sn (*I*4_1_/*amd*) are detected only for *x* = 0.5. The *a*‐, *b*‐, and *c*‐axis lattice parameters (*a*, *b*, and *c*) and the unit cell volume (*V*) of Sn(Se_1−_ *_x_* Te*_x_*) bulks are summarized in Figure 1d. As *x* increases from 0 to 0.5, the *a* increases from 11.50 to 11.79 Å, the *b* increases from 4.15 to 4.28 Å, the *c* increases from 4.44 to 4.56 Å, and thus the *V* is expanded from 212.11 to 230.02 Å^3^, which is explained by the model that the larger Te^2−^ ion substitutes the smaller Se^2−^ site. The *a*, *b*, and *c* values increase linearly (indicated by red lines), while those only for *x* = 0.5 deviates from the straight line, suggesting the solubility limit *x* is a little smaller than 0.5, which is consistent with the SnSe‐SnTe phase diagram (Figure 1a).^[^ ^30^ ^]^ Local structure analyses based on XRD structure refinements for Sn(Se_1−_ *_x_* Te*_x_*) with *x* = 0–0.4 are summarized in Figure S3, Supporting Information, where the Te ion substitution expands all inter‐layer and in‐layer bond distances in SnSe lattice. Note that, to examine the phase stability, the Sn(Se_0.6_Te_0.4_) bulk was annealed at *T* up to 400 °C and then naturally cooled down to RT (Figure S4, Supporting Information). The phase separation to layered SnSe and cubic SnTe phases was observed, supporting the metastability of Sn(Se_1−_ *_x_* Te*_x_*) phase. These results indicate the successful stabilization of the high‐temperature non‐equilibrium layered Sn(Se_1−_ *_x_* Te*_x_*) bulks with *x* up to 0.4.

### 2.2 Electronic Properties

**Figure 2:**
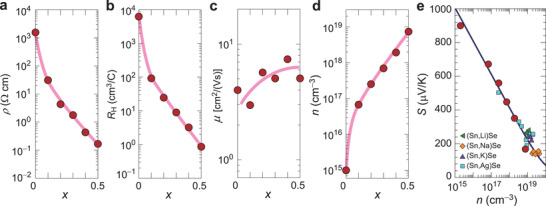
Carrier transport properties of Sn(Se_1−_ *_x_* Te*_x_*) bulks with *x* = 0−0.5 at RT. a) Resistivity, *ρ*, b) Hall coefficient, *R*_H_, c) Hall mobility, *μ*, d) carrier concentration, *n*, and e) Seebeck coefficient, *S*, as a function of *n*. The *S* versus *n* plots of Li, Na, K, and Ag doped SnSe bulks are superimposed for comparison.^[^ ^21^ ^]^

Next, we investigated Te ion substitution effect on electronic properties of Sn(Se_1−_ *_x_* Te*_x_*) bulks. **Figure** **2**a–d summarizes resistivity (*ρ*), Hall coefficient (*R*_H_), Hall mobility (*μ*), and carrier concentration (*n*) for Sn(Se_1−_ *_x_* Te*_x_*) bulks as a function of *x* at RT. The pure SnSe is highly resistive with *ρ* = 1560 Ωcm and the *ρ* largely decreases down to 0.17 Ωcm at *x* = 0.5 (Figure 2a). All the Sn(Se_1−_ *_x_* Te*_x_*) bulks show positive *R*_H_, that is, p‐type hole carriers are dominant (Figure 2b). The *μ* slightly increases from 4 to 5–7 cm^2^ V^−1^s^−1^ with increase of *x*, suggesting that the *μ* at RT is not largely affected by the Te ion substitution (Figure 2c). The large increase of *n* from 1.0 × 10^15^ cm^−3^ (*x* = 0) to 7.4 × 10^18^ cm^−3^ (*x* = 0.5) was observed (Figure 2d). From the *T* dependence of *n* (Figure S5, Supporting Information), the activation energy (*E*_a_) at *T* < 520 K are 0.30 eV for *x* = 0 and 0.01 eV for *x* = 0.4, indicating the high‐density hole generation induces the degenerate conduction in Te‐doped SnSe. Figure 2e shows the *S* versus *n* plots, where those of Li^+^, Na^+^, K^+^, and Ag^+^ doped SnSe bulks are superimposed for comparison.^[^ ^21^ ^]^ The pure SnSe exhibits *S* = +900 μV K^−1^ and the *S* decreases with increase of hole *n*, following the Boltzmann transport theory with the relaxation‐time approximation. The Sn 3d, Se 3d, and Te 3d core level spectra obtained by hard X‐ray photoemission spectroscopy (HAXPES) are shown in Figure S6, Supporting Information, which confirms that Sn, Se, and Te exist as the single valence states each with Sn^2+^, Se^2−^, and Te^2−^, respectively. As will be discussed later, the isovalent Te ion substitution provides the high‐density holes by increasing Sn vacancies in SnSe and acts as simple hole donors similar to aliovalent ion substitution.

### 2.3 Thermoelectric Properties

**Figure 3:**
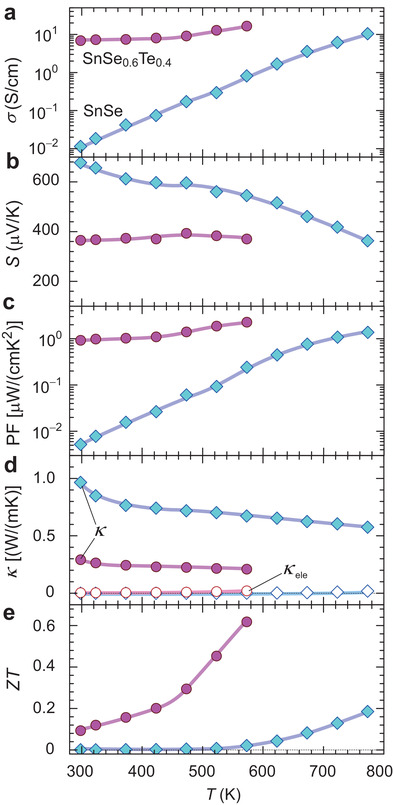
Temperature (*T*) dependences of thermoelectric properties for SnSe and SnSe_0.6_Te_0.4_ bulk polycrystals sintered by SPS. a) Electrical conductivity, *σ*, b) Seebeck coefficient, *S*, c) power factor, PF (=*S* ^2^ *σ*), d) thermal conductivity, *κ*, and e) figure of merit, *ZT*. The electronic *κ* (*κ*_ele_) calculated by the Wiedemann–Franz law is shown in (d).

To investigate the thermoelectric performance of Sn(Se_1−_ *_x_* Te*_x_*), we synthesized high‐density bulk samples of SnSe and Sn(Se_0.6_Te_0.4_) by spark plasma sintering (SPS) under compressive stress 50 MPa and subsequent rapid thermal quenching. By introducing the SPS process, the SnSe and Sn(Se_0.6_Te_0.4_) bulks highly oriented to the *h*00 direction normal to the bulk surface, and the sintering density was largely improved from 78% to 95% (Figure S8, Supporting Information). **Figure** **3**a–e summarizes the *T* dependences of *σ*, *S*, PF (=*S* ^2^ *σ*), *κ*, and *ZT*. *σ* was largely improved by SPS due to the large increases of *μ* up to 20.0 cm^2^ V^−1^s^−1^ for SnSe and 21.8 cm^2^ V^−1^s^−1^ for Sn(Se_0.6_Te_0.4_) bulks at RT. From the *σ–T* (Figure 3a) and the *S–T* (Figure 3b) plots, the SnSe bulk showed the semiconducting *T* dependences, that is, *σ* largely increased and *S* decreased with *T* increase, while the Sn(Se_0.6_Te_0.4_) bulk showed the degenerated conduction, that is, *σ* and *S* were almost unchanged with respect to *T*, due to the high‐density hole generation by the Te ion substitution (Figure 2d). Owing to the large increase of *σ*, the PF was largely improved at wide *T* range below 573 K, where the PF increased from 5.0 × 10^−3^ μW cm^−1^K^−2^ of SnSe to 0.9 μW cm^−1^K^−2^ of Sn(Se_0.6_Te_0.4_) at RT (Figure 3c). In addition, the Te ion substitution largely reduced *κ* from 1.0 W m^−1^K^−1^ of SnSe to 0.3 W m^−1^K^−1^ of Sn(Se_0.6_Te_0.4_) at RT (Figure 3d). In both the cases, the *κ* monotonically decreased with increasing *T*, and the *κ* of Sn(Se_0.6_Te_0.4_) was lower than that of SnSe in the whole *T* range up to 573 K. In order to separate the contribution of *κ*_ele_ and *κ*_lat_ to total *κ*, *κ*_ele_ was estimated by the Wiedemann–Franz law of *κ*_ele_ = *σ* × *L* × *T*, where *L* is the Lorenz number 2.44 × 10^−8^ WΩ K^−2^. Since the contribution of *κ*_ele_ to total *κ* was very small (<7%), Te ion substitution largely reduced *κ*_lat_ of SnSe. *κ*_lat_ depends on the heat capacity (*C*_p_), phonon lifetime (*τ*_*λ*_), phonon group velocity (*v*_*λ*_), and phonon mean free path (*l*_*λ* _= *ν*_*λ* ·_ *τ*_*λ*_) as *κ*_lat_ = 1/3 *C*_p_ × *v*_*λ*_ *× l*_*λ*_. The *C*_p_ = 0.22 J g^−1^K^−1^ of Sn(Se_0.6_Te_0.4_) is slightly smaller than 0.24 J g^−1^K^−1^ of SnSe at RT, and thus the reduction of *κ*_lat_ attributes to the change in the phonon transport parameters of *v*_*λ*_ and/or *τ*_*λ*_. Thus the *ZT* of Sn(Se_0.6_Te_0.4_) was largely improved than that of pure SnSe in all *T* range, resulting in the 30‐times enhancement of *ZT* up to 0.6 at *T* = 573 K (Figure 3e).

Note that the Sn(Se_1−_ *_x_* Te*_x_*) has the anisotropic layered crystal structure, and thus their electronic and thermal transport properties should be measured along the same direction in the bulks. The laser‐flash method can be applied only to a thick bulk sample in order to measure both the in‐plane and the out‐of‐plane *κ*; however, we could not obtain such thick bulk samples because the non‐equilibrium phase could not be stabilized due to the in‐sufficient thermal quenching. Therefore, we measured the in‐plane *κ* of 1‐mm thick Sn(Se_0.6_Te_0.4_) bulk using the steady‐state method only at RT. It provided that the in‐plane *κ*, 0.4 W m^−1^K^−1^, is slightly higher than the out‐of‐plane *κ* (0.3 W m^−1^K^−1^) measured by laser‐flash method at RT. This result indicates that the above *ZT* calculated using the in‐plane *σ* and the out‐of‐plane *κ *should be overestimated (Figure 3e). The measurements of in‐plane *κ *and real *ZT* in Sn(Se_0.6_Te_0.4_) at high *T* will be required in future research, but the present results demonstrate that the isovalent Te ion substitution is an effective approach to enhance PF and reduce *κ* simultaneously to enhance thermoelectric performance in polycrystalline SnSe bulks by the simple synthesis method without nano‐structuring.

### 2.4 Defect Calculations

**Figure 4:**
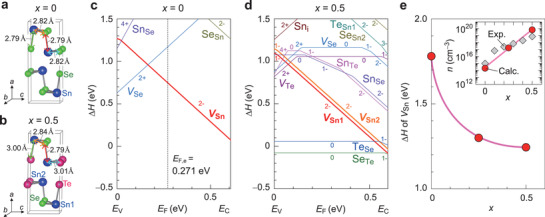
Defect formation analysis of Sn(Se_1−_ *_x_* Te*_x_*). Stable structure models for a) SnSe (*x* = 0) and b) Sn(Se_0.5_Te_0.5_) (*x* = 0.5) drawn by VESTA.^[^ ^32^ ^]^ Defect formation enthalpies (Δ*H*) of defects as a function of Fermi level (*E*_F_) for c) SnSe and d) Sn(Se_0.5_Te_0.5_). Chemical conditions are set at B‐point (Se‐moderate condition) for pure SnSe and A‐point (Se‐poor condition) for Sn(Se_0.5_Te_0.5_) in the chemical potential window in Figure S14, Supporting Information. *E*_F_ is measured from the valence band maximum energy (*E*_V_) and ranges to the conduction band minimum energy (*E*_C_). *V* denotes vacancy, and the subscripts denote defect sites, where i means interstitial sites. The vertical dashed line indicates the equilibrium *E*_F,e_. Note that the *E*_F,e_ for Sn(Se_0.5_Te_0.5_) was −0.070 eV, deeper than *E*_V_. e) Δ*H* of tin vacancy (*V*_Sn_) with the neutral state as a function of *x*. The inset shows the calculated hole carrier concentrations at *E*_F,e_, where the experimental data (Figure 2d) is superimposed for compassion.

To understand the effects of the isovalent Te ion substitution on the hole carrier generation in SnSe, we performed density functional theory (DFT) calculations on the defect formation enthalpies (Δ*H*) using Sn(Se_1−_ *_x_* Te*_x_*) 1 × 3 × 3 supercell models (72 atoms) with *x* = 0, 0.25, and 0.5, in which an ordered Se/Te arrangement was applied (**Figure** **4**a,b; Figure S11, Supporting Information). Figure 4c,d compares the Δ*H* of the defects for vacancies (*V*_Sn_, *V*_Se_, and *V*_Te_), antisites (Sn_Se_, Sn_Te_, Se_Sn_, Se_Te_, Te_Sn_, and Te_Se_), and interstitials (Sn_i_, Se_i_, and Te_i_) as a function of Fermi level (*E*_F_) for SnSe and Sn(Se_0.5_Te_0.5_). Detailed information of the defect calculations are described in Section S6, Supporting Information. For SnSe (*x* = 0), the *V*_Sn_ is the most easily formed defect at high *E*_F_ working as an acceptor, while *V*_Se_ is the one at low *E*_F_ acting as a donor, yielding the hole concentration of 2.14 × 10^14^ cm^−3^ that is almost consistent with the experimentally obtained 1.0 × 10^15^ cm^−3^ of SnSe bulk at *T* = 300 K (Figure 2d). The equilibrium Fermi level (*E*_F,e_) was calculated to be 0.271 eV from the valence band maximum (VBM) energy (*E*_V_) by the charge neutrality condition. For Sn(Se_0.5_Te_0.5_), the Δ*H* of *V*_Sn_ becomes lower than pure SnSe and the *E*_F,e_ was largely shifted to the inside of VBM (*E*_F,e_ = −0.070 eV), which is consistent with the degenerate conduction of Sn(Se_0.6_Te_0.4_). Note that the Δ*H* of Se_Te_ is negative and that of Te_Se_ is small at ≈0.1 eV, while Δ*H* of *V*_Se_ and *V*_Te_ donors at *E*_F,e_ are much high >1.0 eV. This suggests that Se and Te atoms prefer to randomly occupying their sites, and thus *V*_Se_/*V*_Te_ are hardly formed which largely suppresses the electron generation. Figure 4e summarizes the *x* dependence of Δ*H* of *V*_Sn_ at the neutral state, where we take the values from the lowest Δ*H* of *V*_Sn_ sites for Sn(Se_0.75_Te_0.25_) and Sn(Se_0.5_Te_0.5_). With increasing *x* from 0 to 0.5, the Δ*H* of *V*_Sn_ decreases significantly, and the hole concentration largely increases up to ≈10^19^ cm^−3^ (inset of Figure 4e), which is consistent with the experimental result. To further check the mechanism of the easier formation of *V*_Sn_ by Te ion substitution, we performed the chemical bonding analysis for the Sn—Se/—Te bonds of the stable structures of SnSe and Sn(Se_0.5_Te_0.5_) using the crystal orbital Hamiltonian overlap (COHP),^[^ ^33^ ^]^ calculated by LOBSTER code (Figure S19, Supporting Information).^[^ ^34^ ^]^ The Sn—Te bond length (≈3.0 Å) is longer than ≈2.8 Å of Sn—Se bonds in Sn(Se_0.5_Te_0.5_) (Figure 4b), resulting in the weaker bonding strength, that is, the −iCOHP (negative value of integrated COHP) values of 2.57−2.76 eV/bond for Sn—Se bonds in SnSe decrease to 2.37−2.44 eV/bond for Sn—Te bonds in Sn(Se_0.5_Te_0.5_). These results explain that the hole generation originates from the easy formation of *V*_Sn_ due to the weak Sn—Te bonds formed in Sn(Se_1−_ *_x_* Te*_x_*).

### 2.5 Phonon Transport Calculations

**Figure 5:**
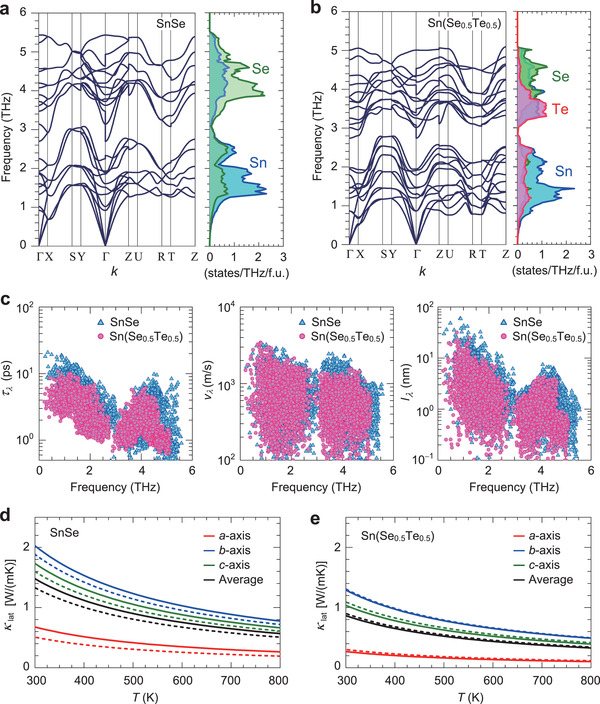
Phonon transport calculations of SnSe and Sn(Se_0.5_Te_0.5_). Phonon band structures (left) and partial phonon density of states (DOSs) of Sn, Se, and Te (right) for a) SnSe and b) Sn(Se_0.5_Te_0.5_). The Γ, X, S, Y, Z, U, R, and T denote the first Brillouin zone internal coordinates, (0, 0, 0), (0.5, 0, 0), (0.5, 0.5, 0), (0, 0.5, 0), (0, 0, 0.5), (0.5, 0, 0.5), (0.5, 0.5, 0.5), and (0, 0.5, 0.5), respectively. c) Phonon life time, *τ*_*λ*_ (left), group velocity, *ν*_*λ*_ (middle), and mean free path, *l*_*λ*_ (right), as a function of phonon frequency for SnSe (blue triangles) and Sn(Se_0.5_Te_0.5_) (pink circles). Temperature (*T*) dependence of calculated *κ*_lat_ along *a*‐ (red solid line), *b*‐ (blue solid line), and *c*‐axes (green solid line) for d) SnSe and e) Sn(Se_0.5_Te_0.5_). The *κ*_lat_ averaged for the *a*‐, *b*‐, and *c*‐axes are shown by the black lines. The dashed lines show the simulated *κ*_lat_ with virtual ion masses, that is, for SnSe the mass of a half of Se ions is replaced by Te mass (d), and for Sn(Se_0.5_Te_0.5_) the mass of all Te ions is replaced by Se mass (e) by keeping the crystal structures.

Finally, in order to elucidate the mechanism for lowering *κ*_lat_ of SnSe by the Te ion substitution, we performed the phonon transport calculations by solving the Peierls–Boltzmann transport equation within the relaxation time approximation, as implemented in the ALAMODE code.^[^ ^35^ ^]^ **Figure** **5**a,b compares the phonon band structures (left) and phonon density of states (DOSs) projected on each element (right) of SnSe and Sn(Se_0.5_Te_0.5_). The phonon dispersion of SnSe (Figure 5a) is characterized by the dispersive acoustic and optical branches along the Г–*Y* (*b*‐axis) and Г–*Z* (*c*‐axis), indicating the significant *v* of optical phonons which is as large as that of acoustic phonons along the in‐layer directions of the SnSe structure. On the other hand, the optical phonon branches are quite flat along the Г–*X* direction (*a*‐axis), which correspond to small *v* and are attributed to weak interactions between adjacent SnSe layers. For Sn(Se_0.5_Te_0.5_) (Figure 5b), the acoustic and optical phonon branches are shifted down, resulting in the weaker phonon dispersion and smaller *v*. Figure 5c compares the phonon properties of *τ*_*λ*_, *ν*_*λ*_, and *l*_*λ* _(=*ν*_*λ* _ × *τ*_*λ*_) with respect to the phonon frequency for SnSe and Sn(Se_0.5_Te_0.5_). As discussed above, the *ν*_*λ* _is reduced in Sn(Se_0.5_Te_0.5_). In addition, compared to SnSe, the Sn(Se_0.5_Te_0.5_) possesses shorter *τ*_*λ* _ especially in the 0–3 and 4–5 THz frequency range, suggesting the stronger phonon scattering. As the result, the *l*_*λ* _(= *ν*_*λ*_ ×*τ*_*λ*_) is decreased for Sn(Se_0.5_Te_0.5_) in the phonon frequency range.

Figure 5d,e compares the *T* dependence of calculated *κ*_lat_ of SnSe and Sn(Se_0.5_Te_0.5_). For both of the SnSe and the Sn(Se_0.5_Te_0.5_) phases, the calculated *κ*_lat_ is quite anisotropic due to the layered structure, that is, the *κ*_lat_ is much smaller along the *a*‐axis than those along the *b*‐ and the *c*‐axes. Along the *a*‐axis, the *κ*_lat_ is 0.28 W m^−1^K^−1^ at *T* = 300 K for Sn(Se_0.5_Te_0.5_) which is almost half of 0.69 W m^−1^K^−1^ for SnSe. The similar trend is seen for *b*‐ and *c*‐axes; the *κ*_lat_ of 1.26 (*b*‐axis) and 1.01 W m^−1^K^−1^ (*c*‐axis) for Sn(Se_0.5_Te_0.5_) are smaller than 1.72 (*b*‐axis) and 2.02 W m^−1^K^−1^ (*c*‐axis) for SnSe. Thus, the *κ*_lat_ averaged for the *a*‐, *b*‐, and *c*‐axes is reduced from 1.47 to 0.85 W m^−1^K^−1^ by the Te ion substitution. In order to further analyze the dominant contribution to low *κ*_lat_ from the heavier mass of Te ion or the weak Sn—Te bonds, we simulated *κ*_lat_ with virtual ion masses, that is, for SnSe the mass of a half of the Se ions is replaced by the Te mass (dotted lines in Figure 5d), and for Sn(Se_0.5_Te_0.5_) where the mass of all Te ions is replaced by Se mass (dotted lines in Figure 5e) by keeping the crystal structures. For SnSe, using the larger Te mass for a half Se ions reduces *κ*_lat_ a little, but the resulting *κ*_lat_ is still almost double of that of Sn(Se_0.5_Te_0.5_). While for Sn(Se_0.5_Te_0.5_), using the smaller Se mass for Te ions shows slight increase compared to that of the original Sn(Se_0.5_Te_0.5_). Thus, the effect of heavier Te mass on the *κ*_lat_ is not large, suggesting that the crystal structure difference especially of the weak Sn—Te bonds largely contributes to the altered lattice vibration (smaller phonon frequency and *ν*_*λ*_) and phonon scattering (lower *τ*_*λ*_), resulting in the *κ*_lat_ reduction of SnSe substituted by Te ions.

## 3 Conclusion

In summary, the nonequilibrium Sn(Se_1−_ *_x_* Te*_x_*) solid solution bulks with *x* up to 0.4 were synthesized by the two‐step process composed of high‐temperature solid‐state reaction and rapid thermal quenching. The Te ion substitution in SnSe realized high *σ* due to the 10^3^‐times increase of hole carrier concentration and effectively reduced *κ*_lat_ from 1.0 W m^−1^K^−1^ of SnSe to 0.3 W m^−1^K^−1^ of Sn(Se_0.6_Te_0.4_), resulting in the large *ZT* enhancement up to 0.6 at 573 K in Sn(Se_0.6_Te_0.4_) bulks although this *ZT* value would be a bit overestimated due to the mixed use of the in‐plane *σ* and the out‐of‐plane *κ*. The weak Sn—Te bonds formed in Sn(Se_1−_ *_x_* Te*_x_*) lattice doped with large‐size Te ion not only increase the hole‐donating Sn vacancies but also reduce the phonon velocities and enhances phonon scattering. Therefore, the isovalent Te ion substitution demonstrated not only optimizing PF but also the well‐reduction of *κ*_lat_ of SnSe, which would give a new guiding principle to enhance the thermoelectric performance of polycrystalline SnSe.

## 4 Experimental Section

### Bulk Synthesis

First, the intermediate compounds SnSe and SnTe were synthesized by solid‐state reactions. High‐purity Sn rods (purity: 99.999%), Se grains (purity: 99.999%), and Te grains (purity: 99.999%) were used as starting reagents. The Sn rods were powdered with a metal file and the Se and Te grains were ground. The Sn, Se, and Te powders were mixed at the molar ratios of Sn:Se = 1.0:1.0 and Sn:Te = 1.0:1.0, and then each mixture was sealed in an evacuated silica‐glass ampule. The sealed ampules for SnSe and SnTe were annealed at 500 °C for 12 h. The synthesized powders were reground and sealed in evacuated silica‐glass ampules. The ampules were heated again at 500 °C for 12 h. The resulting single‐phase SnSe and SnTe powders were mixed at the molar ratios of SnSe:SnTe = 1−*x*:*x*. The mixture was pressed into disks and sealed in an Ar‐filled silica‐glass ampule (before sealing the ampule, the inside of the silica‐glass was vacuumed and then Ar gas was filled at ≈1 atm at RT). Then the ampule was annealed at 700 °C and then subjected to rapid quenching from 700 °C to RT in iced water in order to stabilize the high‐temperature layered Sn(Se_1−_ *_x_* Te*_x_*) solid solution phase. To investigate the thermoelectric properties, the SnSe and SnSe_0.6_Te_0.4_ bulk polycrystals were sintered by SPS. The SnSe and the mixture powders of SnSe and SnTe were pressed into pellets at 400 °C under 50 MPa in vacuum. After that, the sintered pellet was sealed in an Ar‐filled silica‐glass ampule and annealed at 700 °C, and then it was subjected to rapid quenching from 700 °C to RT in iced water. The reagents and products were handled in a glove box filled with a dry Ar gas (the dew point <−80°C, the oxygen concentration < 1 ppm).

### Crystal Structure and Chemical Analysis

Crystalline phases were determined by XRD with the Bragg−Brentano geometry with a Cu K*α* radiation source at RT. The lattice parameters were determined by the Pawley method using the TOPAS ver. 4.2 program (Karlsruhe, Germany: Bruker AXS GmbH). Rietveld analysis, where the fundamental parameter (FP) method was employed, was performed for crystal structure refinement. Chemical compositions of the bulk samples were measured with XRF. The core level spectra were measured by HAXPES at the BL15XU undulator beamline (the excitation X‐ray energy, *hv* = 5953.4 eV) of SPring‐8 at RT. The morphology of the samples was evaluated using a field‐emission scanning electron microscopy (JSM‐7600F, JEOL).

### Electronic and Thermoelectric Property

Electronic properties at RT were measured by Hall effect using the van der Pauw method. Pt electrodes were used for Ohmic contacts. The *σ* and *S* measurements at high *T* were performed along in‐plane direction in the bulk under He atmosphere (ZEM‐3, ADVANCE RIKO). The *κ* was obtained from *κ* = *D* × *C*_p_ × *d*, where the thermal diffusivity (*D*) was measured along out‐of‐plane direction in the bulk under an Ar atmosphere by a laser flash diffusivity method (LFA 457, NETZSCH), the heat capacity (*C*_p_) was measured by differential scanning calorimetry (DSC3500 Sirius, NETZSCH), and the sample density (*d*) was determined by the dimensions and mass of the samples.

### DFT Calculation

The calculations of the band structures, the DOSs, and the defect formation enthalpies for Sn(Se_1−_ *_x_* Te*_x_*) were performed by DFT, conducted using the projector augmented wave method as implemented in the Vienna Ab initio Simulation Package.^[^ ^36^, ^37^ ^]^ Sn [5s5p], Se [4d4p], and Te [5d5p] electronic states were included as valence states. For each stoichiometric Te‐doped SnSe, variable‐cell structure relaxations were performed with the GGA‐PBE functional.^[^ ^38^ ^]^ The plane wave cut‐off energy was set to 600 eV and a *Γ*‐centered 6 × 14 × 14 *k*‐mesh was applied. The phonon calculations including thermal conductivity were performed with the ALAMODE codes.^[^ ^35^ ^]^ Details of calculation methods for defect formation and phonon transport are described in Sections S6,S8, Supporting Information.

## Conflict of Interest

The authors declare no conflict of interest.

## Supporting information

Supporting Information

## Data Availability

The data that support the findings of this study are available from the corresponding author upon reasonable request.
