# Supplementary material for: Degenerated Hole Doping and Ultra‐Low Lattice Thermal Conductivity in Polycrystalline SnSe by Nonequilibrium Isovalent Te Substitution
Source: Adv Sci (Weinh). 2022 Mar 8;9(13):2105958. doi: 10.1002/advs.202105958 (PMC9069380; doi:10.1002/advs.202105958)
Supplement: Supplementary file 1 — Supporting Information [file ADVS-9-2105958-s001.pdf]

ADVANCED SCIENCE
Open Access

Supporting Information

for *Adv. Sci.*, DOI 10.1002/advs.202105958

Degenerated Hole Doping and Ultra-Low Lattice Thermal Conductivity in Polycrystalline SnSe by Nonequilibrium Isovalent Te Substitution

Xinyi He, Haoyun Zhang, Takumi Nose, Takayoshi Katase, Terumasa Tadano, Keisuke Ide, Shigenori Ueda, Hidenori Hiramatsu, Hideo Hosono and Toshio Kamiya**

© 2022 Wiley-VCH GmbH

Supporting Information and Figures S1–S23

Degenerated hole doping and ultra-low lattice thermal conductivity in polycrystalline SnSe by nonequilibrium isovalent Te substitution

Xinyi He, Haoyun Zhang, Takumi Nose, Takayoshi Katase, Terumasa Tadano, Keisuke Ide, Shigenori Ueda, Hidenori Hiramatsu, Hideo Hosono, and Toshio Kamiya**

Section 1. Crystal structure and chemical composition analyses of $Sn(Se_{1-x}Te_x)$ bulks
Section 2. Phase stability of $Sn(Se_{0.6}Te_{0.4})$ bulks
Section 3. Carrier transport properties and electronic structure analysis of $Sn(Se_{1-x}Te_x)$ bulks
Section 4. Effect of spark plasma sintering of $Sn(Se_{1-x}Te_x)$ bulks
Section 5. Electronic structure calculation of $Sn(Se_{1-x}Te_x)$ bulks
Section 6. Chemical potential window and defect calculations of $Sn(Se_{1-x}Te_x)$
Section 7. Chemical bonding analysis of $Sn(Se_{1-x}Te_x)$
Section 8. Phonon transport calculations of $Sn(Se_{1-x}Te_x)$

Supporting Information

Section 1. Crystal structure and chemical composition analyses of Sn($Se_{1-x}Te_x$) bulks

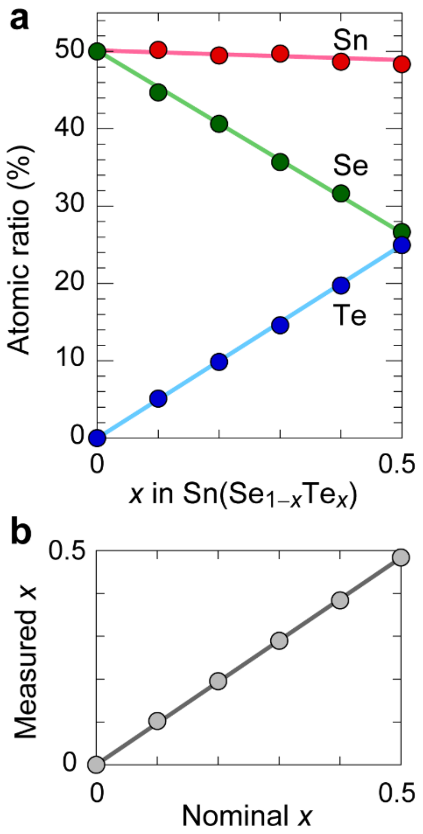

Figure S1. (a) Atomic compositions for Sn($Se_{1-x}Te_x$) bulks measured by XRF with respect to the nominal *x*. (b) Relationship between nominal *x* and measured *x* for Sn($Se_{1-x}Te_x$).

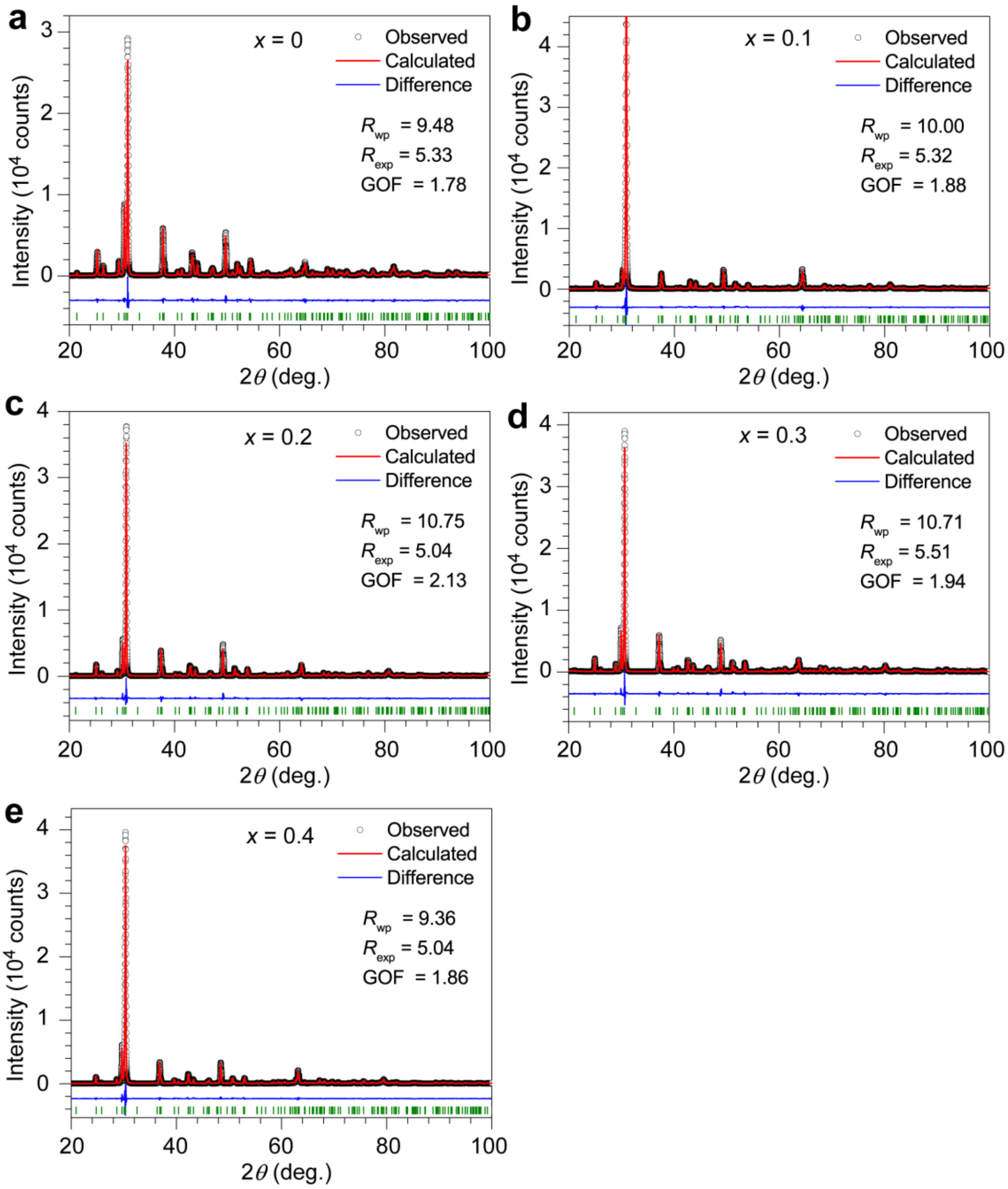

Figure S2. Rietveld analysis for $Sn(Se_{1-x}Te_x)$ bulk polycrystals with (a) $x = 0$, (b) 0.1, (c) 0.2, (d) 0.3, and (e) 0.4. The green vertical bars in each figure indicate the diffraction angles of the layered SnSe phase (space group: *Pnma*).

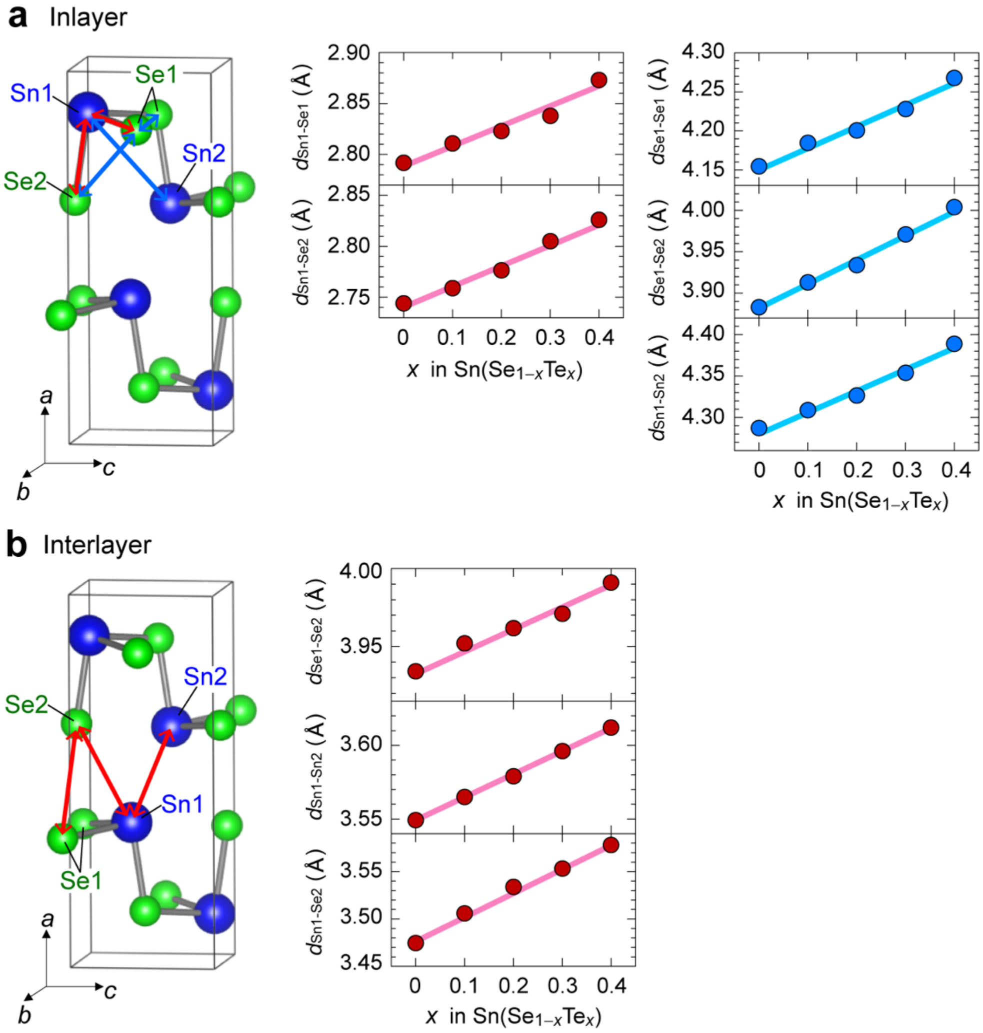

Figure S3. (a) Intra-layer bond distances and (b) inter-layer bond distances for $Sn(Se_{1-x}Te_x)$ obtained by Rietveld analysis for XRD patterns (Fig. S2).

Section 2. Phase stability of Sn($Se_{0.6}Te_{0.4}$) bulks

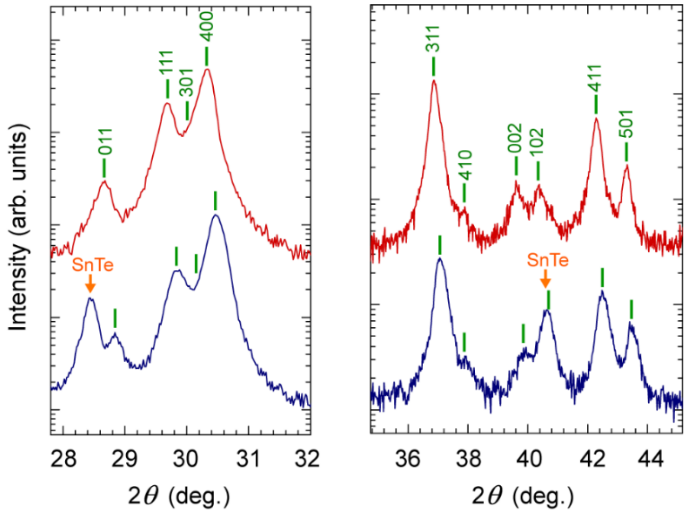

Figure S4. XRD patterns of Sn($Se_{0.6}Te_{0.4}$) bulks annealed at 300 °C (top) and 400 °C (bottom) for 2 hours and then naturally cooled down to RT in Ar atmosphere. The green vertical bars in each figure indicate the diffraction angles of layered SnSe phase (space group: *Pnma*). Single phase of layered Sn($Se_{0.6}Te_{0.4}$) was observed for 300 °C annealed sample, but cubic SnTe phase appeared for 400 °C annealed one, as indicated by orange arrows. The lattice parameters were estimated to be a = 11.7467 Å, b = 4.2528 Å, and c = 4.5327 Å for 300 °C annealed sample, and a = 11.7213 Å, b = 4.2402 Å, c = 4.5214 Å for 400 °C annealed one. The cubic SnTe impurity phase has a = 6.2684 Å. The appearance of cubic SnTe phase and the decrease of lattice parameters of layered SnSe phase indicate that phase separation from non-equilibrium layered Sn($Se_{1-x}Te_x$) solid-solution to a mixuture of layered SnSe and cubic SnTe phases occurs at 400 °C.

Section 3. Carrier transport properties and electronic structure analysis of Sn($Se_{1-x}Te_x$) bulks

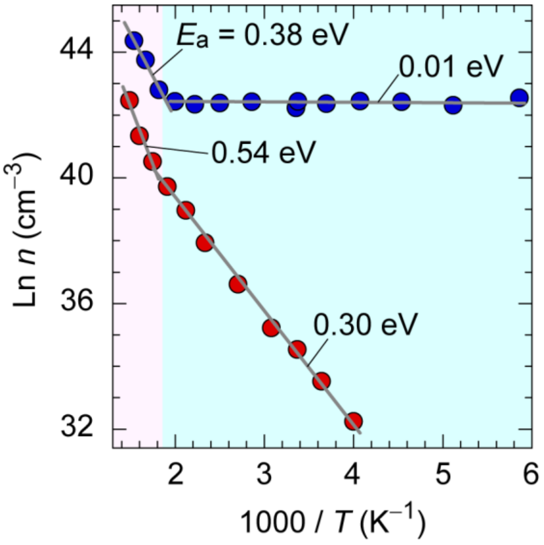

Figure S5. Arrhenius plots of carrier concentration n, $\ln(n) = \left(-\frac{E_a}{k_B}\right)\left(\frac{1}{T}\right) + \ln(n_0)$, where E_a and k_B are activation energy and the Boltzmann constant, respectively, measured by Hall effect for SnSe (red plots) and $SnSe_{0.6}Te_{0.4}$ (blue plots) bulk polycrystals. Each curve has two different slopes in ln(n) vs. 1/T plots. The E_a of n in low T region < 520 K are 0.30 eV for x = 0 and 0.01 eV for x = 0.4, indicating the heavy hole generation provides the degenerate conduction of SnSe by Te ion substitution. The E_a of n in the high T region > 520 K are 0.54 eV for x = 0 and 0.38 eV for 0.4, which are almost a half of the bandgaps obtained by diffuse reflectance (**Fig. S7**), suggesting this range is in the intrinsic T region of semiconductor and supporting that the estimation of bandgap of Sn($Se_{1-x}Te_x$) is reasonable.

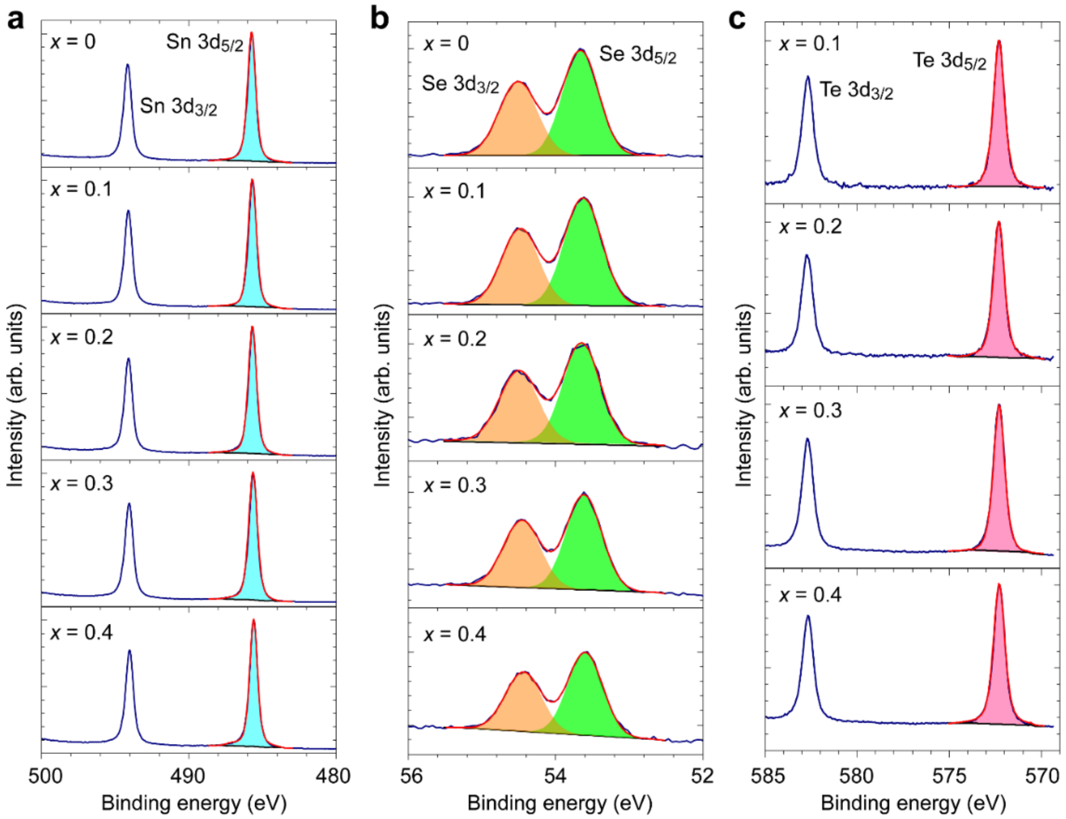

Figure S6. (a) Sn 3d, (b) Se 3d, and (c) Te 3d core level hard X-ray photoemission spectroscopy (HAXPES) spectra for Sn($Se_{1-x}Te_x$) bulks with x = 0–0.4 at RT. There are two peaks in each Sn 3d, Se 3d, and Te 3d core spectrum, corresponding to the spin-orbit-splitting of $3d_{5/2}$ and $3d_{3/2}$ levels. The Sn $3d_{5/2}$ and Te $3d_{5/2}$ core level spectra are fitted by single symmetric peak each. The Se 3d core level spectrum is fitted by two symmetric peaks for Se $3d_{3/2}$ peak (shaded with orange) and Se $3d_{5/2}$ (shaded with green). The black lines and the red lines indicate the background and total fitting curves, respectively.

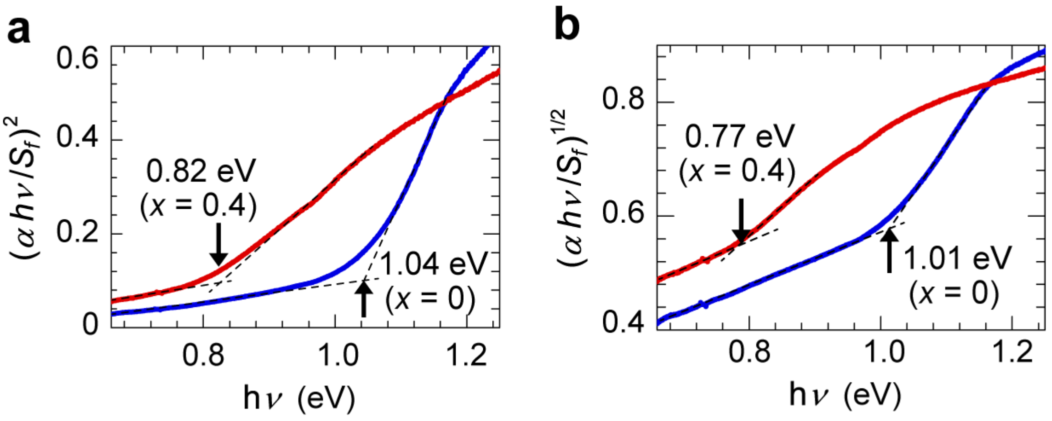

Figure S7. Kubelka-Munk plots of (a) $(\alpha h\nu/S_f)^2$ for direct gap and (b) $(\alpha h\nu/S_f)^{1/2}$ for indirect gap, where α, S_f, h, and ν respectively denote the optical absorption coefficient, the scattering factor, the Planck constant, and the frequency, for SnSe and $SnSe_{0.6}Te_{0.4}$ bulk polycrystals. For pure SnSe, the estimated bandgap is 1.01 eV (indirect) and 1.04 eV (direct). Meanwhile the bandgaps for $SnSe_{0.6}Te_{0.4}$ are 0.77 eV (indirect) and 0.82 eV (direct). For both cases, the indirect gaps are smaller than the direct gap, and the bandgap becomes smaller by Te ion substitution.

Section 4. Effect of spark plasma sintering of $Sn(Se_{1-x}Te_x)$ bulks

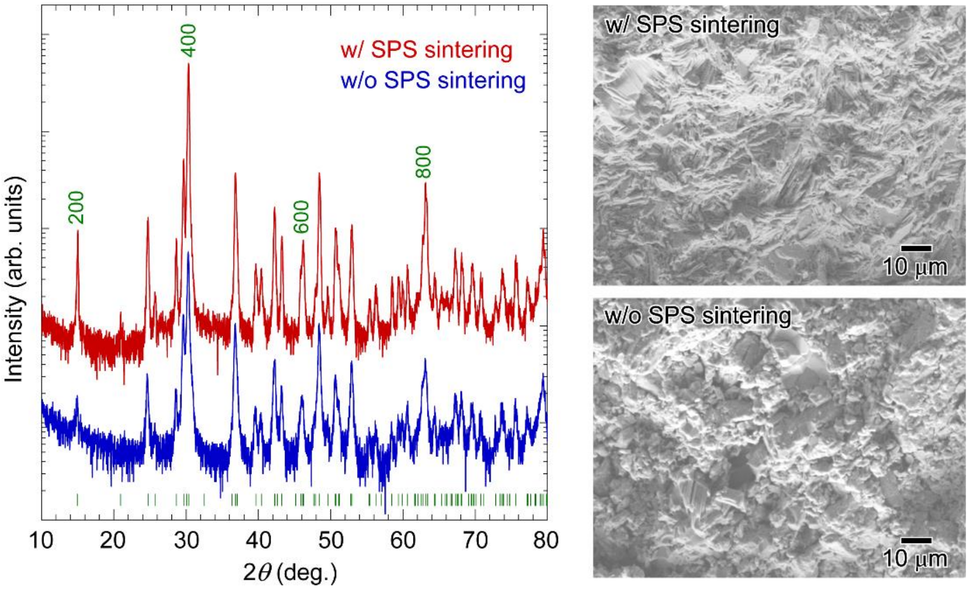

Figure S8. XRD patterns (left) and cross-sectional FE-SEM images (right) of $Sn(Se_{0.6}Te_{0.4})$ bulks synthesized without and with SPS. The green vertical bars in left figure indicate the diffraction angles of the layered SnSe phase (space group: *Pnma*). The SPS process strongly enhanced the *h*00 preferential orientation along the out-of-plane direction of the bulk and highly densified the $Sn(Se_{0.6}Te_{0.4})$ planar crystals.

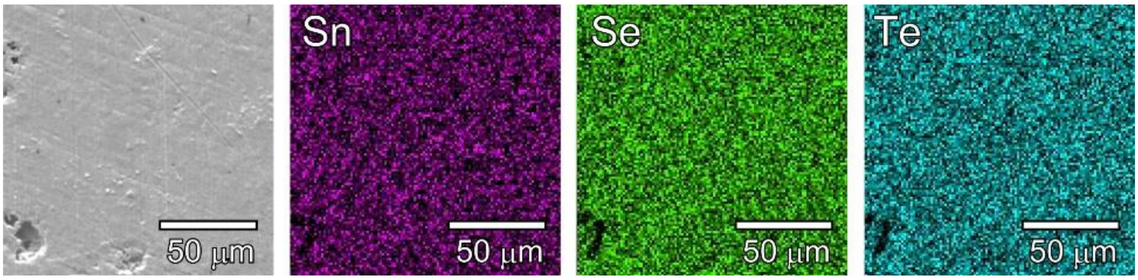

Figure S9. FE-SEM image and energy dispersive X-ray spectroscopy (EDS) mappings of Sn, Se, and Te elements for the polished surface of $SnSe_{0.6}Te_{0.4}$ bulk polycrystal synthesized with SPS.

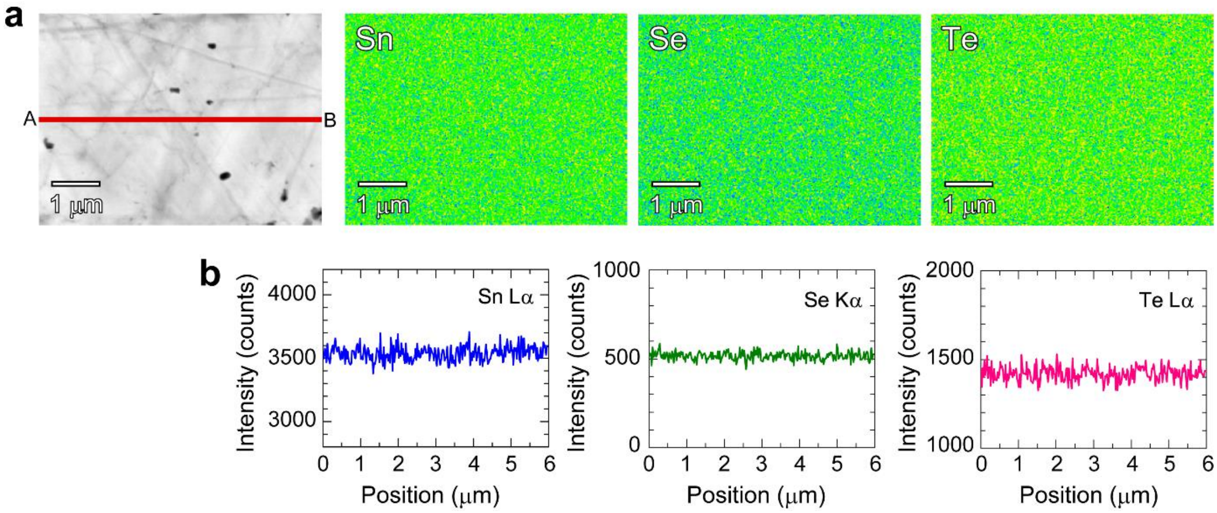

Figure S10. (a) High magnification EPMA chemical composition mappings of Sn, Se, and Te elements for the polished surface of $SnSe_{0.6}Te_{0.4}$ bulk polycrystal synthesized with SPS. (b) Chemical composition line profiles between A–B in (a) for Sn, Se, and Te elements. The chemical composition mappings of Sn, Se, and Te clearly support the uniformity in the whole region of the bulk.

Section 5. Electronic structure calculation of Sn($Se_{1-x}Te_x$) bulks

The relaxed crystal structures of SnSe, $SnSe_{0.75}Te_{0.25}$, and $SnSe_{0.5}Te_{0.5}$ were obtained by DFT with GGA-PBE functional[1] (cut off energy: 600eV, Γ-centered *k*-mesh: 6×14×14). The stable structures are shown in **Fig. S11**, where the Se sites are substituted by Te ions. The *a*, *b*, *c*, and *V* calculated for the Sn($Se_{1-x}Te_x$) primitive cell models are summarized in **Fig. S12**. Considering that the ground-state lattice parameters by DFT typically include errors within 2–3%, the calculated results are consistent with the experimental data within the variation of the functionals. This trend was confirmed also by PBEsol functional[2]. The gradient for the variations in *a*, *b*, *c*, and *V* (blue solid lines) with respect to the composition *x* is almost consistent with the experimental ones (red line) at *x* up to 0.4. That is, the model, where the Se sites are substituted by Te ions, explains the experimental structures well, and strongly supports that the Te dopants are successfully incorporated into the Se sites in the SnSe lattice.

Figures S13(a,b) compare the electronic band structures of SnSe and Sn($Se_{0.5}Te_{0.5}$), calculated by DFT with GGA-PBE functional, where the energy levels are aligned to the deep Se 4s energy levels. Both phases have indirect bandgap and the valence band maximum (VBM) shifts to higher energy by Te ion substitution, leading to the reduced bandgap, which is consistent with the experimental result of indirect gap reduction from 1.01 eV of SnSe bulk to 0.77 eV of Sn($Se_{0.6}Te_{0.4}$) bulk, measured by diffuse reflectance (**Fig. S7**). The total density of states (DOSs) and partial DOSs (pDOSs) projected on each element for SnSe and Sn($Se_{0.5}Te_{0.5}$) are summarized in **Figs. S13(c,d).** The valence band of SnSe is formed by the hybridization of Sn 5s / 5p orbitals and Se 4p orbitals, while for Sn($Se_{0.5}Te_{0.5}$) the shallower Te 5p orbitals additionally contribute to the VBM, which pushes up the valence band edge.

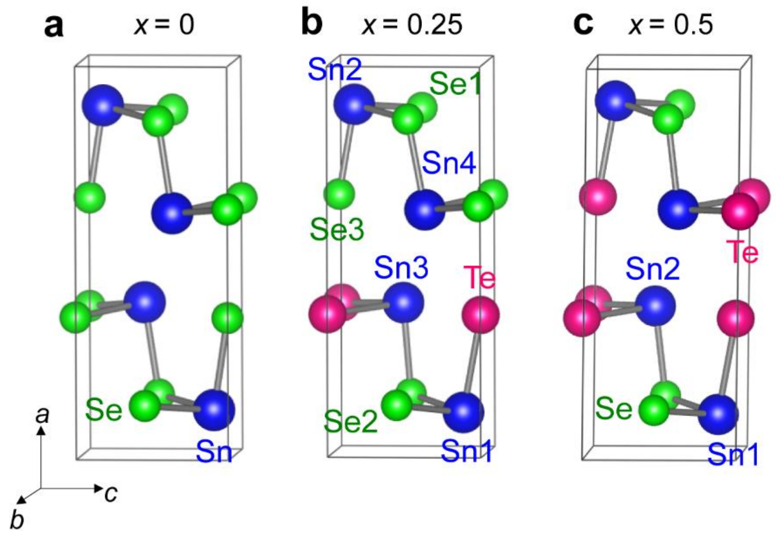

Figure S11. Relaxed crystal structures of (a) pure SnSe, (b) $Sn(Se_{0.75}Te_{0.25})$, and (c) $Sn(Se_{0.5}Te_{0.5})$ models obtained by DFT calculations with GGA-PBE functional.

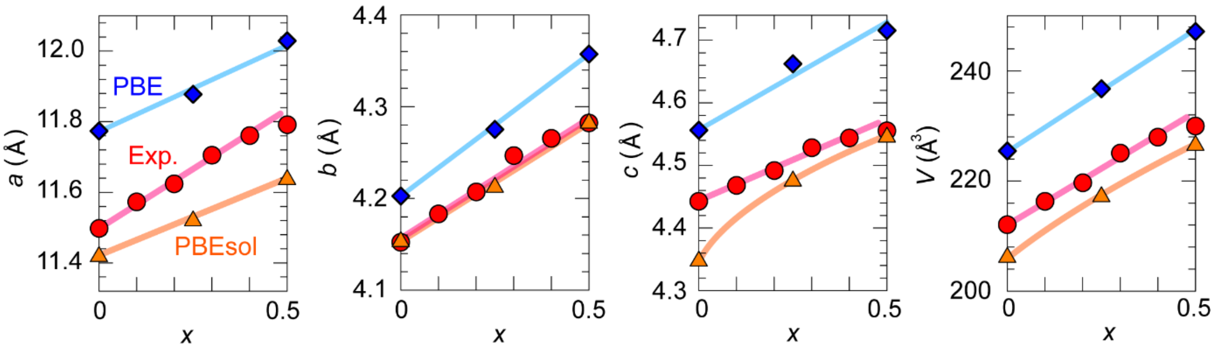

Figure S12. The *a*-axis, *b*-axis, and *c*-axis lattice parameters (*a*, *b*, *c*) and unit cell volume (*V*) calculated by GGA-PBE functional (blue diamonds) and PBEsol functional (orange triangles) for $Sn(Se_{1-x}Te_x)$ with x = 0, 0.25, and 0.5. The experimentally obtained *a*, *b*, *c*, and *V* (red circles) for $Sn(Se_{1-x}Te_x)$ bulks are shown for comparison.

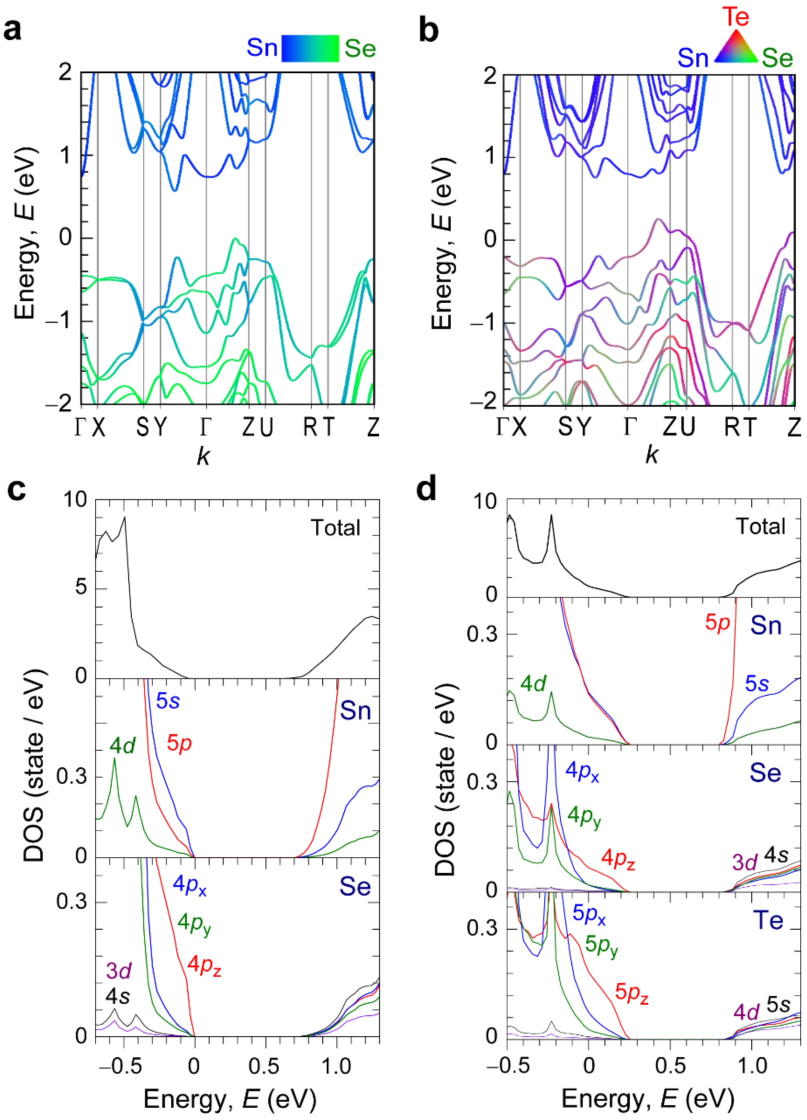

Figure S13. Electronic structures of Sn($Se_{1-x}Te_x$). (a,b) Electronic band structures of SnSe (a) and Sn($Se_{1-x}Te_x$) with x = 0.5 (b), calculated with GGA-PBE functional. The energy is aligned to the Se 4s levels (not shown). The Γ, X, S, Y, Z, U, R, and T denote the first Brillouin zone internal coordinates (0, 0, 0), (0.5, 0, 0), (0.5, 0.5, 0), (0, 0.5, 0), (0, 0, 0.5), (0.5, 0, 0.5), (0.5, 0.5, 0.5), and (0, 0.5, 0.5), respectively. (c,d) Total density of state (DOS) and partial DOSs projected on each element of Sn, Se, and Te.

Section 6. Chemical potential window and defect calculations of Sn($Se_{1-x}Te_x$)

Chemical potential window calculations. The chemical potential windows of SnSe, Sn($Se_{0.75}Te_{0.25}$), and Sn($Se_{0.5}Te_{0.5}$) were calculated from the total energies of layered Sn($Se_{1-x}Te_x$) phase and possible other phases with the relaxed crystal structure by GGA-PBE functional. In order to stabilize the Sn($Se_{1-x}Te_x$) phase, the following thermodynamic equilibrium equations should be satisfied.

$$\Delta\mu_{\mathrm{Sn}} + \Delta\mu_{\mathrm{Se}} = \Delta H_{\mathrm{SnSe}} = -0.88\ \mathrm{eV} \quad (1)$$

$$\Delta\mu_{\mathrm{Sn}} + 0.75\Delta\mu_{\mathrm{Se}} + 0.25\Delta\mu_{\mathrm{Te}} = \Delta H_{\mathrm{SnSe0.75Te0.25}} = -0.77\ \mathrm{eV} \quad (2)$$

$$\Delta\mu_{\mathrm{Sn}} + 0.5\Delta\mu_{\mathrm{Se}} + 0.5\Delta\mu_{\mathrm{Se}} = \Delta H_{\mathrm{SnSe0.5Te0.5}} = -0.68\ \mathrm{eV} \quad (3)$$

, where ΔH_{SnSe}, $\Delta H_{\mathrm{SnSe0.75Te0.25}}$, and $\Delta H_{\mathrm{SnSe0.5Te0.5}}$ is the formation enthalpy of SnSe, $SnSe_{0.75}Te_{0.25}$ and $SnSe_{0.5}Te_{0.5}$, as referred to elemental Sn solid (face-centered cubic, $Fm\bar{3}m$), Se solid (monoclinic, $P21/c$) and Te solid (trigonal, $P3_121$). The chemical potentials of Sn, Se, and Te must be less than those of the elementary phases to avoid the formation of the Sn, Se or Te elemental phases, thus $\Delta\mu_{\mathrm{Sn}} < 0$, $\Delta\mu_{\mathrm{Se}} < 0$, and $\Delta\mu_{\mathrm{Te}} < 0$ are required. Furthermore, to suppress the formation of the competing secondary phases $SnSe_2$ (trigonal, $P3m1$),[3] and SnTe (cubic, $Fm\bar{3}m$),[4] the following conditions must be satisfied as well.

$$\Delta\mu_{\mathrm{Sn}} + 2\Delta\mu_{\mathrm{Se}} < \Delta H_{\mathrm{SnSe_2}} = -1.06\ \mathrm{eV} \quad (4)$$

$$\Delta\mu_{\mathrm{Sn}} + \Delta\mu_{\mathrm{Te}} < \Delta H_{\mathrm{SnTe}} = -0.59\ \mathrm{eV} \quad (5)$$

Since the high temperature layered $SnSe_{0.75}Te_{0.25}$ and $SnSe_{0.5}Te_{0.5}$ phases are obtained under the nonequilibrium thermodynamic condition, there are no chemical potential windows for these Te-doped phases. So in order to get the chemical potential windows for the following defect formation energy calculations, we slightly decreased the formation enthalpy of $SnSe_{0.75}Te_{0.25}$ and $SnSe_{0.5}Te_{0.5}$ phases by 0.02 eV/atom and 0.03 eV/atom, respectively. Under these constrains, $\Delta\mu_{\mathrm{Sn}}$, $\Delta\mu_{\mathrm{Se}}$ and $\Delta\mu_{\mathrm{Te}}$ that stabilize the layered Sn($Se_{1-x}Te_x$) phase are restricted to the red area as shown in **Fig. S14**, drawn with CHESTA.[5]

Defect calculations. For the defect formation enthalpy (ΔH) calculations, a 72-atoms supercell with 1×3×3 primitive cells and a Γ-centered 3×3×3 k-mesh were used, in which atomic internal coordinates were fully relaxed by GGA-PBE functional until all the forces on the atoms became less than 0.03 eV/Å and the total energy difference was smaller than 10^{-4} eV with the fixed cell parameters so as to meet the dilute limit condition. The corresponding plane wave cut-off energy for the basis was set to 250 eV. The convergence test of the cut-off energy was shown in **Fig. S15**. The difference in total energy between the cut-off energy 250 eV and 500 eV is within 1 meV/atom, which is much smaller than the calculation accuracy of VASP (~10 meV). We considered the intrinsic point defects in $Sn(Se_{1-x}Te_x)$ phase, including vacancies (V_{Sn}, V_{Se}, and V_{Te}), cation substitutions at the Sn site (Se_{Sn}, Te_{Sn}), anion substitutions at Se site (Sn_{Se}, Te_{Se}), and interstitials (Sn_i, Se_i, Te_i).

The defect formation energy in the charge state q ($\Delta H_{D,q}$) is defined as: [6]

$$\Delta H_{D,q} = E_D - E_h + q(E_V + E_F) + \sum n_i\mu_i + E_{corr.} \qquad (6)$$

, where E_D is the total energy of the supercell with the defect in the charge state q, and E_h is that of the perfect host supercell. The (E_V + E_F) is the additional energy of electrons trapped by the defect at E_F. n_i indicates the number of the i-th atoms added ($n_i < 0$) or removed ($n_i > 0$), and μ_i is the chemical potential of the i-th atom with respect to that of the corresponding elemental phase (μ_i^{el}) by $\mu_i = \mu_i^{el} + \Delta\mu_i$. $E_{corr.}$ is the correction value for the defect formation enthalpy in order to suppress the considerable supercell finite-size effects. The standard correction methods for finite-size effects were applied, including the potential-alignment correction and the image charge correction[6] to get the accurate value of the total energies for the charged defect-containing models.

The convergence test of the formation energy of V_{Sn}^{2-} as a function of the supercell size is shown in **Fig. S16** for the reason that V_{Sn} is the most important defect in $Sn(Se_{1-x}Te_x)$ and the −2 charge state is affected most seriously by the charge interaction in the finite supercell.

The largest size of supercell is limited to 2×5×5 supercell containing 400 atoms due to the computational constraints. The origins of the relative energy below are taken at the zero corrected energy of the largest supercell. It shows that the difference between the corrected energy of the 1×3×3 supercell and the largest 2×5×5 supercell is within 0.1 eV, which guarantees that the 1×3×3 supercell provides satisfactory converged values.

For quantitative analysis, the equilibrium E_F ($E_{F,e}$) at T_D were calculated by solving semiconductor statistic equations. The defect concentrations $c_{D,q}$ are calculated by the statistic equation:[7]

$$c_{D,q}(E_F, \mu, T_D) = N_{site,D} \exp\left(\frac{\Delta S_{D,q}}{k_B} - \frac{\Delta H_{D,q}(E_F,\mu)}{k_B T_D}\right)/Z \quad (7)$$

$$Z = \sum_{D',q'} \exp\left(\frac{\Delta S_{D',q'}}{k_B} - \frac{\Delta H_{D',q'}(E_F,\mu)}{k_B T_D}\right) \quad (7')$$

, where Z is the partition function and $N_{site,D}$ is the concentration of the possible sites/configurations where the defect D can be formed, k_B is the Boltzmann constant, $\Delta S_{D,q}$ is the formation entropy (we employed the constant entropy approximation with the value of $10k_B$.),[7-10] and T_D is the defect frozen temperature (we take 300 K for pure SnSe and 973.15 K for Te-doped SnSe due to the rapid quenching process which makes the defects frozen in the high temperature state). The $E_{F,e}$, $c_{D,q}$ for given μ and T_D and the electron concentration (n) in the conduction band and the hole concentration (p) in the valence band were determined so as to satisfy the charge neutrality condition at 300K. The $E_{F,e}$ at RT for given μ and T_D were determined so as to satisfy the charge neutrality condition. **Figure S17** summarizes the ΔH of defects in SnSe, $Sn(Se_{0.75}Te_{0.25})$, and $Sn(Se_{0.5}Te_{0.5})$ as a function of E_F at the chemical potential conditions of A point (Sn-rich/Se-poor chemical condition), B point (Sn-moderate/Se-moderate chemical condition), and C point (Sn-poor/Se-rich chemical condition) in the chemical potential window (**Fig. S14**). The calculated hole concentrations of SnSe as a function of E_F are shown in **Fig. S18**. The chemical potential condition of pure

SnSe was set at Se-moderate condition (B-point in **Fig. S14**) in order that the calculated hole concentration of 2.14×10^{14} cm^{-3} is consistent with the experimentally obtained ~ 1×10^{15} cm^{-3} of SnSe bulk. Note that at $E_{F,e}$, the hole concentrations produced by V_{Sn}^{2-} is 1.07×10^{14} cm^{-3} and it is slightly compensated by the electrons produced by V_{Se} (8.28×10^{6} cm^{-3}), thus, yielding the hole concentration of 2.14×10^{14} cm^{-3}. On the othra hand, the chemical potential condition of $Sn(Se_{0.5}Te_{0.5})$ was set at Se-poor condition (A-point in **Fig. S14**) because the Sn impurities appeared in the XRD patterns (**Fig. 1(a)**).

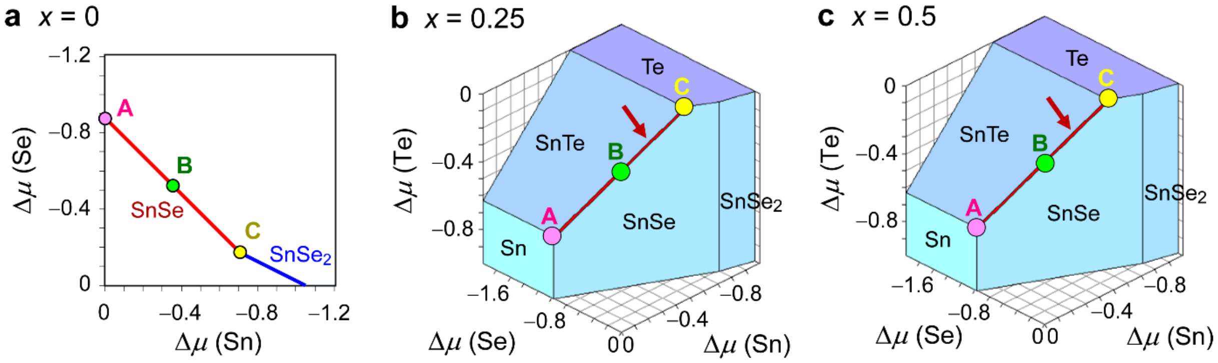

Figure S14. Calculated chemical potential window of (a) SnSe, (b) $Sn(Se_{0.75}Te_{0.25})$, (c) $Sn(Se_{0.5}Te_{0.5})$, as a function of the chemical potentials of Sn ($\Delta\mu_{Sn}$ with respect to elemental Sn), Se ($\Delta\mu_{Se}$), and Te ($\Delta\mu_{Te}$), visualized with CHESTA[5]. The line A-B-C shows the stable region boundary of SnSe and $Sn(Se_{1-x}Te_{x})$ phase against possible competitive phases. The A points are Sn-rich/Se-poor chemical conditions, B points are Sn-moderate/Se-moderate chemical conditions, and C points are Sn-poor/Se-rich chemical conditions.

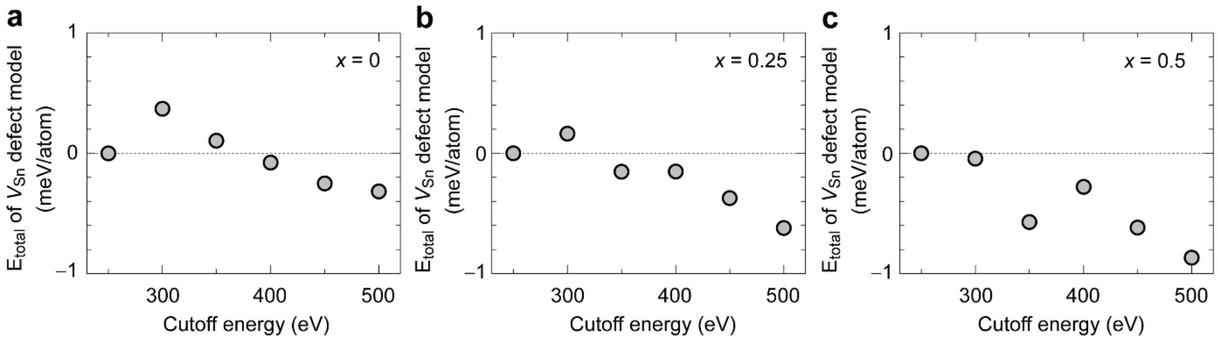

Figure S15. The convergence test in terms of the cut-off energy for the total energy (E_{total}) of V_{Sn}^{2-} in (a) SnSe, (b) $Sn(Se_{0.75}Te_{0.25})$, and (c) $Sn(Se_{0.5}Te_{0.5})$.

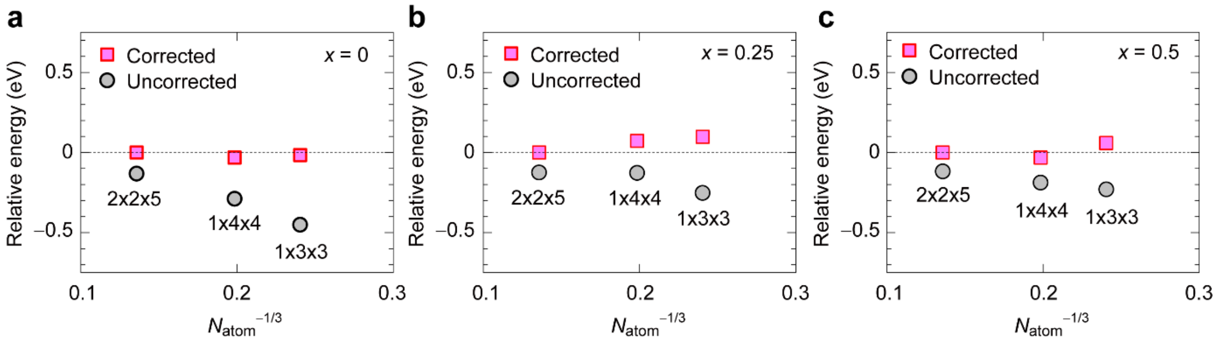

Figure S16. The uncorrected and the corrected relative formation energies of V_{Sn}^{2-} as a function of the supercell sizes in (a) SnSe, (b) $Sn(Se_{0.75}Te_{0.25})$, and (c) $Sn(Se_{0.5}Te_{0.5})$. The origins of the relative energy below are taken at the zero corrected energy of the largest supercell.

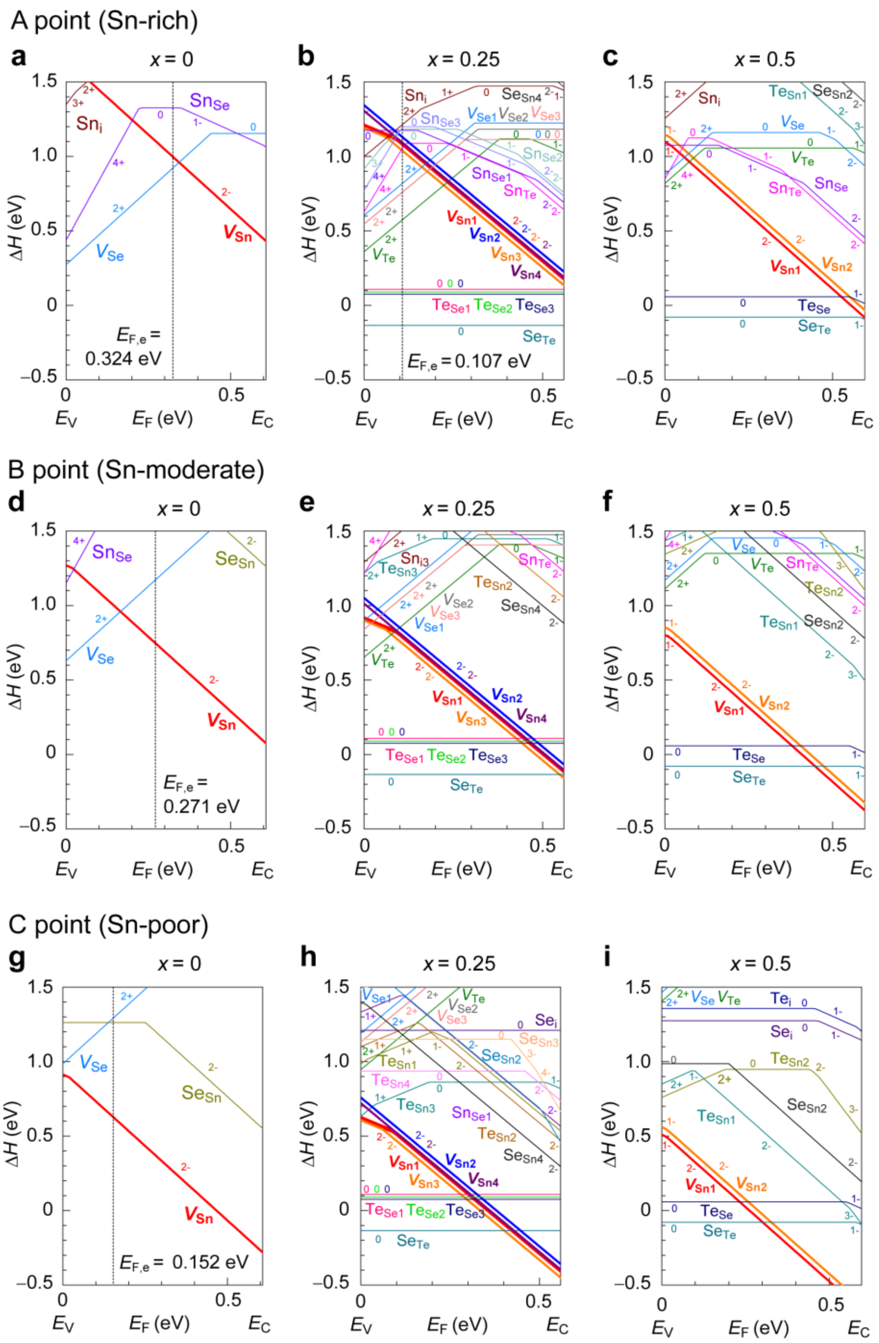

Figure S17. Defect formation enthalpies (ΔH) of defects in (a,d,g) SnSe, (b,e,h) $Sn(Se_{0.75}Te_{0.25})$, and (c,f,i) $Sn(Se_{0.5}Te_{0.5})$ as a function of Fermi level (E_F). Chemical potential conditions are set at A point (Sn-rich/Se-poor chemical condition) for (a-c), B point (Sn-moderate/Se-moderate chemical condition) for (d-f), C point (Sn-poor/Se-rich chemical condition) for (d-i) in the chemical potential window (**Fig. S14**). E_F is measured from valence band maximum (E_V) and ranges to conduction band minimum (E_C). The V denotes vacancy, and the subscripts denote defect sites, where i means interstitial sites. The vertical dashed

lines mark the equilibrium $E_{F,e}$.

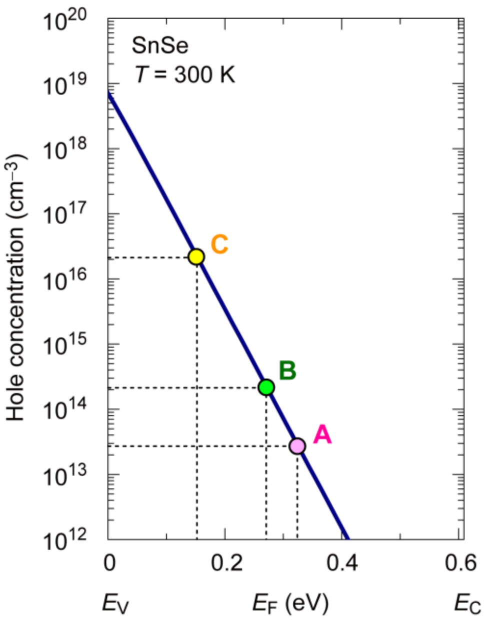

Figure S18. Calculated hole concentration as a function of Fermi level (E_F) at A point (Sn-rich/Se-poor chemical condition), B point (Sn-moderate/Se-moderate chemical condition), and C point (Sn-poor/Se-rich chemical condition) for SnSe. The hole concentration of 2.14×10^{14} cm^{-3} at the B point in the chemical potential window (**Fig. S14**) is consistent with experimentally obtained 1.0×10^{15} cm^{-3} of SnSe bulk.

Section 7. Chemical bonding analysis of Sn($Se_{1-x}Te_x$)

Chemical bonding analysis. The chemical bonding analysis for the Sn-Se/Te bonds of SnSe and Sn($Se_{0.5}Te_{0.5}$) was performed by using the crystal orbital Hamiltonian overlap (COHP),[11] calculated by LOBSTER codes.[12] **Figure S19** summarizes the bond length and the –iCOHP value (the integrated –COHP up to E_F, to estimate the bonding strength) of Sn-Se/Te bonds. In the pure SnSe, the inter-layer Sn-Se bond (Sn1-Se2 and Sn2-Se3 bond in **Fig. S19(c)**) possesses the shortest bond length than the in-layer Sn-Se bond (Sn1-Se1 and Sn2-Se2 bond in **Fig. S19(c)**), corresponding to the largest –iCOHP value. By introducing the Te ions, the Sn-Te bonds have longer bond length than Sn-Se bonds, in which, the inter-layer Sn2-Te3 bond expands from 2.79 Å to 3.00 Å, while the in-layer Sn1-Te1 bond expands from 2.82 Å to 3.01 Å, so that the –iCOHP value of the inter-layer Sn2-Te/Se3 bond decreases from 2.76 eV/bond for SnSe to 2.44 eV/bond for Sn($Se_{0.5}Te_{0.5}$), and the value of the in-layer Sn1-Te/Se1 bond also becomes smaller from 2.57 eV/bond for SnSe to 2.37 eV/bond for Sn($Se_{0.5}Te_{0.5}$). This variation is consistent with the result that V_{Sn} is more easily formed in Sn($Se_{0.5}Te_{0.5}$) with the longer bond distance and weaker bond strength for Sn-Te bond. Note that the different bond dissociation energy for Sn-Te (= 338.1 kJ/mol) is smaller than than for Sn-Se (= 401.2 kJ/mol),[13] which also expects that the introduction of Te ions in SnSe lattice leads to the easier V_{Sn} formation. Actually, the SnTe shows much high $p > 10^{20}$ cm^{-3} due to intrinsic V_{Sn}.[14,15]

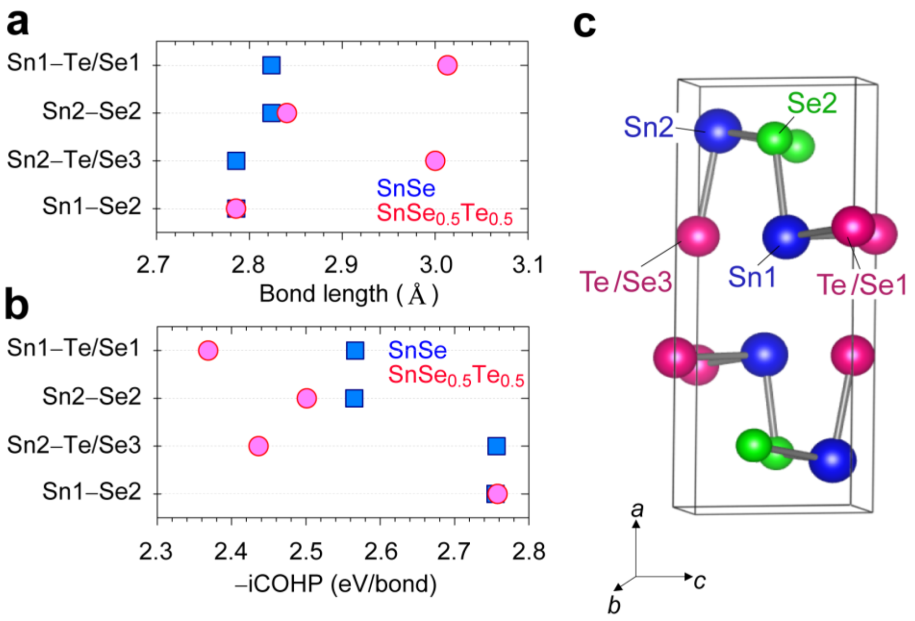

Figure S19. (a) Bonding length and (b) calculated –iCOHPs for the Sn-Se/Te bonding in the crystal structure (c) for SnSe and $SnSe_{0.5}Te_{0.5}$.

Section 6. Phonon transport calculations of Sn($Se_{1-x}Te_x$)

Phonon transport calculations. The phonon transport calculations were performed by solving the Peierls–Boltzmann transport equation within the relaxation time approximation, as implemented in the ALAMODE code.[16] For Sn($Se_{1-x}Te_x$), a 2×3×3 supercell (144 atoms) with a Γ-centered 2×3×3 *k*-mesh was used for the harmonic interatomic force constants (IFCs), and a 1×2×2 supercell (32 atoms) with a Γ-centered 3×4×4 *k*-mesh was used for the anharmonic 3rd-order IFCs. The harmonic IFCs were fixed to the values determined by the finite-displacement approach.[17,18] We included all allowed interactions for the harmonic IFCs and the 3rd-order IFCs inside the cutoff radii of 12 bohr. The DFT calculations to obtain the force were performed using the PBEsol functional with a plane-wave energy cutoff of 400 eV, a convergence criterion for the electronic self-consistency loop of 10^{-8} eV, and the Gaussian smearing method with a smearing width of 0.05 eV. The non-analytic correction was included to the dynamical matrix by the mixed-space approach[19] with the Born effective charges of constituent elements and dielectric constant calculated by density functional perturbation theory.[20] The lattice thermal conductivity (κ_{lat}) was calculated by solving the Boltzmann transport equation under the single-mode relaxation time approximation with a 5×13×13 *q* point mesh. The convergence test in terms of the *q*-meshes is described in **Fig. S20**, which confirms the above *q*-mesh has enough accuracy. Phonon-isotope scatterings are considered for both phases.[21]

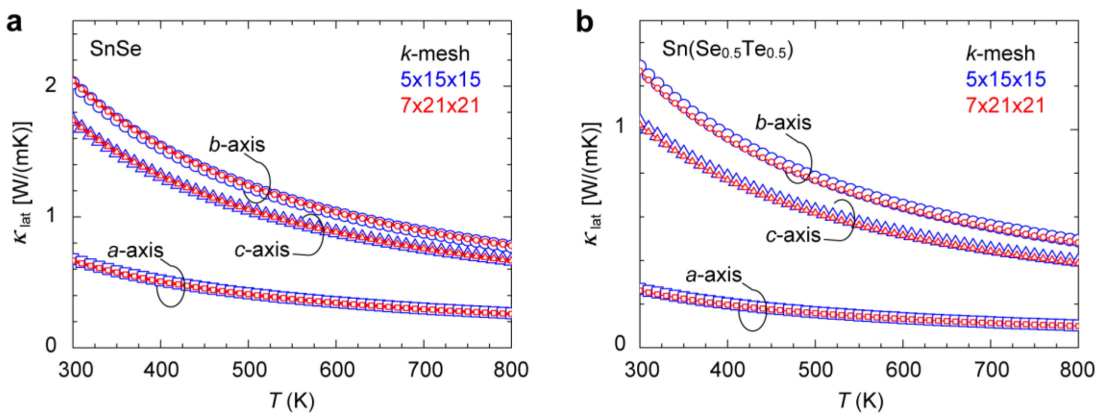

Figure S20. The convergence test in terms of the *q*-meshes for the temperature (*T*) dependence of κ_{lat} along *a*-, *b*-, and *c*-axes of (a) SnSe and (b) $Sn(Se_{0.5}Te_{0.5})$.

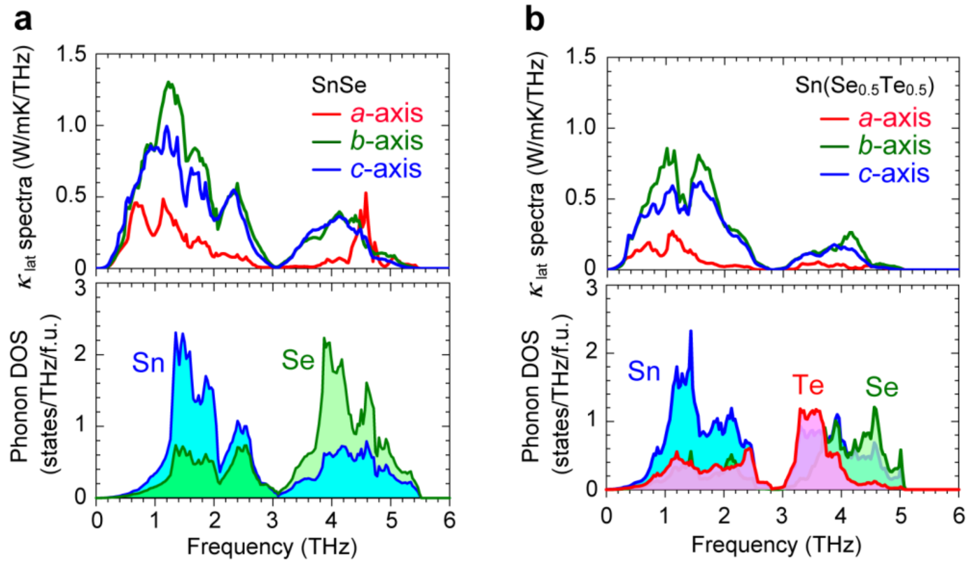

Figure S21. The κ_{lat} spectra (top panel) and atom-projected phonon partial density of states (DOSs) of Sn, Se, and Te with respect to the phonon frequency (bottom panel) for (a) SnSe and (b) $Sn(Se_{0.5}Te_{0.5})$. The phonon DOSs are separated into two major regimes; the lower and higher frequency parts are mainly contributed by the vibrations of Sn and Se/Te, respectively. For both of them, the low-frequency phonon (< 3 THz) occupy a larger part of contribution to κ_{lat} for all directions.

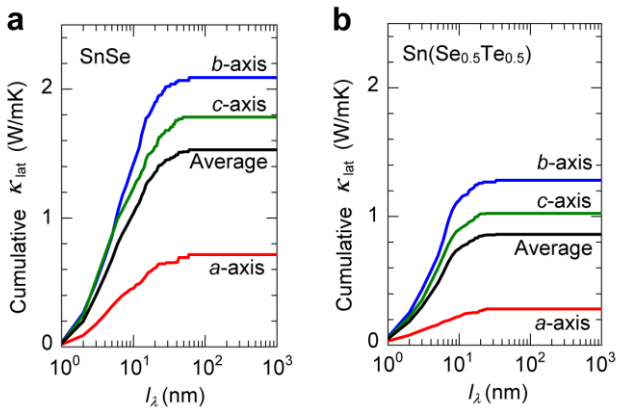

Figure S22. Cumulative κ_{lat} along *a*-, *b*-, and *c*-axes direction with respect to phonon mean free path, l_λ for (a) SnSe and (b) $Sn(Se_{0.5}Te_{0.5})$ at T = 300 K. The cumulative κ_{lat} averaged for all axis is shown by black line. For both cases, the κ_{lat} are dominated by phonons with short l_λ of 1–30 nm and the l_λ corresponding to 50% κ_{lat} accumulation are only ~10 nm for pure SnSe and ~5 nm for $Sn(Se_{0.5}Te_{0.5})$. Since the crystal sizes of polycrystalline SnSe and $Sn(Se_{0.6}Te_{0.4})$ are large > 10μm (**Fig. S8**), the effect of l_λ to experimental κ_{lat} would be negligible.

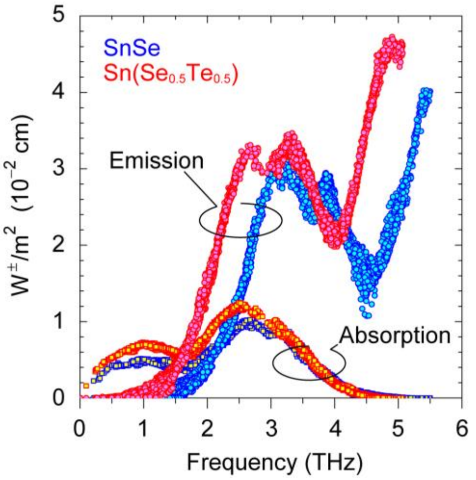

Figure S23. The calculated scattering phase space ($W^{\pm}/m^2$) at T = 300 K for SnSe and $Sn(Se_{0.5}Te_{0.5})$. The phonon scattering emission part largely increases in the 1.5~3 THz and 4~5 THz frequency range for the $Sn(Se_{0.5}Te_{0.5})$. Note that the phonon frequency range, where the phonon scattering emission part increases, overlaps with the decrease in τ_λ (**Fig. 5(c)**), indicating the phonon scattering channels is increased in SnSe by Te ion substitution.

References

[1] J. P. Perdew, K. Burke, M. Ernzerhof, *Phys. Rev. Lett.* **1996**, *77*, 3865–3868.

[2] P. Perdew, A. Ruzsinszky, G. I. Csonka, O. A. Vydrov, G. E. Scuseria, L. A. Constantin, X. Zhou, K. Burke, *Phys. Rev. Lett.* **2008**, *100*, 136406.

[3] A. J. Smith, P. E. Meek, W. Y. Liang. *J. Phys. C: Solid State Phys.* **1977**, *10*, 1321–1333.

[4] M. Le Bouteiller, A. M. Martre, R. Farhi, C. Petot, *Metall. Mater. Trans. B* **1977**, *8*, 339–344.

[5] N. Hatada, http://www.aqua.mtl.kyoto-u.ac.jp/chesta.html.

[6] S. Lany, A. Zunger, *Phys. Rev. B* **2008**, *78*, 235104.

[7] D. B. Laks, C. G. Van de Walle, G. F. Neumark, P. E. Blöchl S. T. Pantelides, *Phys. Rev. B* **1992**, *45*, 10965–10978.

[8] J. Neugebauer, C.G. Van de Walle, *Advances in Solid State Physics* **1996,** *35*, 25–44.

[9] Z. Xiao, F.-Y. Ran, H. Hosono, T. Kamiya, *Appl. Phys. Lett.* **2015**, *106*, 152103.

[10] X. He, T. Katase, K. Ide, H. Hosono, T. Kamiya, *Inorg. Chem.* **2021**, 60 (14), 10227–10234.

[11] R. Dronskowski, P. E. Bloechl, *J. Phys. Chem.* **1993**, *97*, 8617–8624.

[12] S. Maintz, V. L. Deringer, A. L. Tchougréeff, R. Dronskowski, *J. Comput. Chem.* **2016**, *37*, 1030–1035.

[13] Y.-R. Luo, Comprehensive Handbook of Chemical Bond Energies, CRC Press, Boca Raton, FL, **2007**.

[14] R. Brebrick, *J. Phys. Chem. Solids* **1963**, *24*, 27–36.

[15] R. Brebrick, A. Strauss, *Phys. Rev.* **1963**, *131*, 104.

[16] T. Tadano, Y. Gohda, S. Tsuneyuki, *J. Phys.: Condens. Matter* **2014**, *26*, 225402.

[17] G. Kresse, J. Furthmüller, J. Hafner, *Europhys. Lett.* **1995**, *32*, 729−734.

[18] K. Parlinski, Z. Li, Y. Kawazoe, *Phys. Rev. Lett.* **1997**, *78*, 4063−4066.

[19] Y. Wang, J. Wang, W. Wang, Z. Mei, S. Shang, L. Chen, Z. Liu, *J. Phys. Condens. Matter* **2010**, *22*, 202201.

[20] S. Baroni, S. De Gironcoli, A. Dal Corso, P. Giannozzi, *Rev. Mod. Phys.* **2001**, *73*, 515.

[21] S. I. Tamura, *Phys. Rev. B* **1983**, *27*, 858.
